# Diabetes and gastric cancer incidence and mortality in the Asia Cohort Consortium: A pooled analysis of more than a half million participants

**DOI:** 10.1111/1753-0407.13561

**Published:** 2024-05-16

**Authors:** Katherine De la Torre, Minkyo Song, Sarah Krull Abe, Md. Shafiur Rahman, Md. Rashedul Islam, Eiko Saito, Sukhong Min, Dan Huang, Yu Chen, Prakash C. Gupta, Norie Sawada, Akiko Tamakoshi, Xiao‐Ou Shu, Wanqing Wen, Ritsu Sakata, Jeongseon Kim, Chisato Nagata, Hidemi Ito, Sue K. Park, Myung‐Hee Shin, Mangesh S. Pednekar, Shoichiro Tsugane, Takashi Kimura, Yu‐Tang Gao, Hui Cai, Keiko Wada, Isao Oze, Aesun Shin, Yoon‐Ok Ahn, Habibul Ahsan, Paolo Boffetta, Kee Seng Chia, Keitaro Matsuo, You‐Lin Qiao, Nathaniel Rothman, Wei Zheng, Manami Inoue, Daehee Kang

**Affiliations:** ^1^ Department of Biomedical Sciences Seoul National University Graduate School Seoul Korea; ^2^ Department of Preventive Medicine Seoul National University College of Medicine Seoul Korea; ^3^ Infections and Immunoepidemiology Branch, Division of Cancer Epidemiology and Genetics National Cancer Institute Bethesda Maryland USA; ^4^ Laboratory of Epidemiology and Population Sciences National Institute on Aging, National Institute of Health Baltimore Maryland USA; ^5^ Division of Prevention National Cancer Center Institute for Cancer Control Tokyo Japan; ^6^ Research Center for Child Mental Development, Hamamatsu University School of Medicine Tokyo Japan; ^7^ Hitotsubashi Institute for Advanced Study, Hitotsubashi University Tokyo Japan; ^8^ Institute for Global Health Policy Research, National Center for Global Health and Medicine Tokyo Japan; ^9^ Integrated Major in Innovative Medical Science, Seoul National University Graduate School Seoul Korea; ^10^ Department of Population Health and Environmental Medicine NYU Grossman School of Medicine New York New York USA; ^11^ Healis – Sekhsaria Institute for Public Health Navi Mumbai India; ^12^ Division of Cohort Research National Cancer Center Institute for Cancer Control Tokyo Japan; ^13^ Department of Public Health Hokkaido University Faculty of Medicine Sapporo Japan; ^14^ Division of Epidemiology, Department of Medicine, Vanderbilt Epidemiology Center Vanderbilt‐Ingram Cancer Center Vanderbilt University Medical Center Nashville Tennessee USA; ^15^ Radiation Effects Research Foundation Hiroshima Japan; ^16^ Graduate School of Cancer Science and Policy, National Cancer Center Goyang Korea; ^17^ Department of Epidemiology and Preventive Medicine Gifu University Graduate School of Medicine Gifu Japan; ^18^ Department of Preventive Medicine, Division of Cancer Information and Control Aichi Cancer Center Research Institute Nagoya Japan; ^19^ Division of Descriptive Cancer Epidemiology Nagoya University Graduate School of Medicine Nagoya Japan; ^20^ Department of Social and Preventive Medicine Sungkyunkwan University School of Medicine Seoul Korea; ^21^ International University of Health and Welfare Graduate School Tokyo Japan; ^22^ Department of Epidemiology Shanghai Cancer Institute Shanghai China; ^23^ Renji Hospital, Shanghai Jiaotong University School of Medicine Shanghai China; ^24^ Division of Cancer Epidemiology and Prevention Aichi Cancer Center Research Institute Nagoya Japan; ^25^ Cancer Research Institute, Seoul National University Seoul Korea; ^26^ Department of Public Health Sciences University of Chicago Chicago Illinois USA; ^27^ Stony Brook Cancer Center, Stony Brook University Stony Brook New York USA; ^28^ Department of Medical and Surgical Sciences University of Bologna Bologna Italy; ^29^ Saw Swee Hock School of Public Health, National University of Singapore Singapore Singapore; ^30^ Department of Cancer Epidemiology Nagoya University Graduate School of Medicine Nagoya Nagoya Japan; ^31^ School of Population Medicine and Public Health, Chinese Academy of Medical Sciences and Peking Union Medical College Beijing China; ^32^ Occupational and Environmental Epidemiology Branch, Division of Cancer Epidemiology and Genetics National Cancer Institute Bethesda Maryland USA

**Keywords:** Asia, diabetes, gastric cancer, incidence, mortality, prospective studies

## Abstract

**Background:**

Evidence suggests a possible link between diabetes and gastric cancer risk, but the findings remain inconclusive, with limited studies in the Asian population. We aimed to assess the impact of diabetes and diabetes duration on the development of gastric cancer overall, by anatomical and histological subtypes.

**Methods:**

A pooled analysis was conducted using 12 prospective studies included in the Asia Cohort Consortium. Among 558 981 participants (median age 52), after a median follow‐up of 14.9 years and 10.5 years, 8556 incident primary gastric cancers and 8058 gastric cancer deaths occurred, respectively. Cox proportional hazard regression models were used to estimate study‐specific hazard ratios (HRs) and 95% confidence intervals (CIs) and pooled using random‐effects meta‐analyses.

**Results:**

Diabetes was associated with an increased incidence of overall gastric cancer (HR 1.15, 95% CI 1.06–1.25). The risk association did not differ significantly by sex (women vs men: HR 1.31, 95% CI 1.07–1.60 vs 1.12, 1.01–1.23), anatomical subsites (noncardia vs cardia: 1.14, 1.02–1.28 vs 1.17, 0.77–1.78) and histological subtypes (intestinal vs diffuse: 1.22, 1.02–1.46 vs 1.00, 0.62–1.61). Gastric cancer risk increased significantly during the first decade following diabetes diagnosis (HR 4.70, 95% CI 3.77–5.86), and decreased with time (nonlinear *p* < .01). Positive associations between diabetes and gastric cancer mortality were observed (HR 1.15, 95% CI 1.03–1.28) but attenuated after a 2‐year time lag.

**Conclusion:**

Diabetes was associated with an increased gastric cancer incidence regardless of sex, anatomical subsite, or subtypes of gastric cancer. The risk of gastric cancer was particularly high during the first decade following diabetes diagnosis.

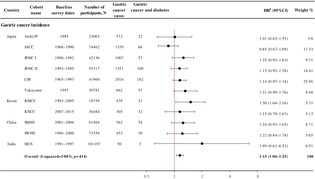

## Graphical abstract

Highlights

- Having diabetes increased gastric cancer risk by 15%, and the risk increased during the first decade following diabetes diagnosis.
- Diabetes increases the risk of gastric cancer regardless of sex, anatomical subsite, or histological subtype.
- Given the substantial diabetes burden in Asia and other regions, our findings underline the importance of diabetes diagnosis and diabetes duration for gastric cancer prevention.

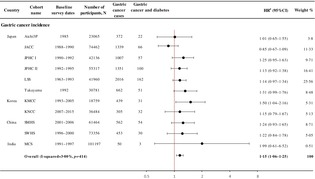

## 1 INTRODUCTION

Asian countries, particularly those in East Asia, have the highest incidence rates of gastric cancer, despite a declined trend in recent years.^1^ Conversely, the prevalence of diabetes is increasing globally, particularly in Asia, where more than 60% of those with the disease reside.^2^ Diabetes is linked to increased cancer risk, including liver, pancreatic, endometrial, colorectal, breast, and bladder cancers, potentially due to hyperinsulinemia, hyperglycemia, and chronic inflammation.^3^ However, evidence linking diabetes and gastric cancer remains inconclusive.

Several studies have found a positive association between diabetes and gastric cancer risk,^4^ whereas some studies reported otherwise.^5^, ^6^ Some reported the association in both men and women.^7^, ^8^ whereas others found the association restricted to women.^9^, ^10^ Only a few have evaluated this association by anatomical subsites of gastric cancer with inconclusive results.^7^, ^11^ In Asia, studies in Korea, Japan, and Hong Kong have reported the association between diabetes and gastric cancer risk with conflicting findings.^8^, ^9^, ^10^, ^11^, ^12^ Limitations of these studies include small number of cases (*n* < 200),^11^, ^12^ inadequate control of potential confounders such as demographic and lifestyle factors,^8^ failure to stratify by sex (gastric cancer incidence rates twice as high in men),^8^, ^11^, ^12^ and insufficient analysis of anatomical or histological subtypes.^8^, ^9^, ^10^, ^12^ Furthermore, although there have been studies incorporating the impact of the diabetes duration,^8^, ^11^ none has explored its dose–response effect.

Diabetes among Asians shows epidemiological characteristics different from the Western population.^13^ It has been hypothesized that Asians may have an early beta‐cell dysfunction and impaired insulin action due to low lean mass.^14^ Consequently, understanding the relationship between diabetes and gastric cancer among Asians may help identify differential risks for this population, potentially leading to better prevention strategies and surveillance guidelines for patients with diabetes. Within the Asia Cohort Consortium,^15^, ^16^ the present study aims to elucidate the association between diabetes and gastric cancer in the Asian population by sex, anatomic subsites, histologic subtypes, and diabetes duration.

## 2 METHODS

### 2.1 Study design and population

The Asia Cohort Consortium is an ongoing international collaborative study designed to investigate the association between genetic and environmental factors and the onset of various diseases and conditions.^17^ Over 1 million healthy participants are included from 40 participating cohorts from 10 Asian countries. For the current study, 12 cohorts agreed to participant from China (Shanghai Men's Health Study [SMHS], Shanghai Women's Health Study [SWHS]), Japan (Three‐Prefecture Cohort Study Aichi [Aichi3P], Japan Collaborative Cohort Study [JACC], Japan Public Health Center‐Based Prospective Study [JPHC I, II]), Life Span Study [LSS], and Takayama Study), Korea (Korea Multicenter Cancer Cohort Study [KMCC], Korea National Cancer Center Cohort Study [KNCC], Seoul Male Cancer Cohort [SeoulM]) and India (Mumbai Cohort Study [MCS]). Details on the cohort‐specific characteristics are provided in Table S1. Data from the participating cohorts were harmonized and deidentified at the Asia Cohort Consortium coordinating center. This study was approved by the ethics committee overseeing each cohort study and by the institutional review board of the Asia Cohort Consortium coordinating centers (Fred Hutchinson Cancer Research Center, Seattle, WA, USA; National Cancer Center, Tokyo, Japan). The study follows the Strengthening the Reporting of Observational Studies in Epidemiology reporting guideline.^18^

All participant cohorts were required to have data on gastric cancer incidence and/or mortality and self‐reported diabetes diagnosis at baseline. We excluded individuals with missing information on age and sex (*n* = 3585), follow‐up time (incidence, *n* = 7850; mortality, *n* = 7684), gastric cancer outcome (incidence, *n* = 17 613; deaths, *n* = 0), diabetes status (*n* = 80 310), and previous diagnoses of any cancer at the baseline (*n* = 8173). After exclusion, the main analyses included 558 981 participants from 11 cohorts for the analysis of incidence and 580 663 from 12 cohorts for mortality (Figures S1 and S2).

### 2.2 Exposure assessment

Data on the self‐reported diagnosis of diabetes were collected at recruitment in each cohort. Previous studies evaluated the validity of self‐reported diabetes in the Asia Cohort Consortium by comparing it with glycosuria results.^19^ Age at diabetes diagnosis was available in five cohorts (KMCC, KNCC, SeoulM, SMHS, and SWHS). Duration of diabetes was calculated using three approaches: (a) sum of the duration of diabetes at recruitment and follow‐up time (ie, diabetes duration at censoring), (b) years between age at diabetes diagnosis and age at enrollment, and (c) restricted to newly diagnosed diabetes (diabetes duration <1 year at enrollment). Information regarding the type of diabetes, blood glucose levels, or medication use was unavailable.

### 2.3 Outcome ascertainment

Ascertainment of cancers through linkage to death certificates, cancer registries, vital statistics registries, or active follow‐up surveys depending on each cohort protocol (Table S1). Gastric cancers were defined based on the *International Classification of Diseases* (ICD), either using the Ninth (code 150) or the Tenth (code C16) Revision.

Gastric cancer anatomical subsite was available in seven cohorts (AichiP3, JACC, JPHC I, and II, LSS, KMCC, KNCC) and categorized as cardia (151.0, C16.0) and noncardia (151.1–151.9, C16.1–C16.9). Gastric cancer histology was available in five cohorts (AichiP3, JACC, JPHC I, and II, LSS) only for incidence analysis, and classified as intestinal (8012, 8021–8022, 8031–8032, 8046, 8050, 8082, 8143–8144, 8201, 8210–8211, 8220–8221, 8255, 8260–8263, 8310, 8323, 8480–8481, 8510, 8512, 8570, and 8576) and diffuse (8020, 8041, 8044, 8141–8142, 8145, 8490, and 8806), based on morphology code of the ICD for Oncology 3 (Lauren classification).

### 2.4 Covariates

Information on demographics, lifestyle factors, and anthropometry (KMCC, KNCC, SMHS, and SWHS measured by trained staff) were collected by questionnaires at enrollment. Body mass index (BMI) was calculated as weight in kilograms divided by height in meters squared (kg/m^2^). Smoking and alcohol consumption was categorized as never or ever, except for the Takayama cohort where the variable “current alcohol consumption” (yes/no) was used. Education was classified as low (less than primary school) and high (middle school or higher). Missing values were imputed using the median for continuous and the mode for categorical variables because the proportion of these missing values were <5% for each cohort.

### 2.5 Statistical analysis

Baseline characteristics were summarized using mean, median, range, and SD for continuous, and percentages for categorical variables. Hazard ratios (HRs) and 95% confidence intervals (CIs) were calculated using Cox proportional hazards regression for each cohort separately, using age as the time metric, and pooled by random‐effects meta‐analysis. Time at entry was defined as the age at enrollment. Time at exit was either age at first primary gastric cancer diagnosis (incidence) or age at gastric cancer death (mortality), age at any‐cause death, loss to follow‐up, or age at the end of follow‐up, whichever occurred first. A nonproportional test based on Schoenfeld partial residuals was performed to assess the Cox proportional hazards assumption of predictors constant over time for each cohort. Heterogeneity among cohorts was assessed by the *I* ^2^ index. Models were adjusted for smoking status, alcohol consumption, BMI, and educational level depending on the availability in each cohort (Table S1). Stratified analyses were conducted by sex, country, enrollment year, age at enrollment, age at cancer diagnosis/death, and birth year.

We performed secondary analyses including gastric cancer anatomical subsites and histological subtypes. Additionally, we conducted an analysis using diabetes duration as an exposure variable. Diabetes duration was analyzed by quartiles calculated for each cohort. A dose–response analysis using a random‐effect model was conducted for evaluating linear and nonlinear dose–response relationships.^20^

Sensitivity analyses were performed by excluding gastric cancer cases occurring within the first 2 years of follow‐up and restricting participants >30 years old to reduce likelihood possible inclusion of cases of type 1 diabetes. Additionally, we considered probable diabetes‐related deaths (incidence: 11069; mortality: 11492) occurring before the outcome as competing risks and developed subdistribution hazard models to investigate the impact of diabetes and diabetes duration on gastric cancer risk^21^ (Table S2). Furthermore, pooled meta‐analyses were repeated, omitting each study (leave‐one‐out analysis) for assessing the relative weight of each study. Quantitative probabilistic bias analysis was performed to assess unmeasured confounder bias for *Helicobacter pylori*, a known risk for which data were unavailable.^22^ Based on previous research,^23^, ^24^, ^25^ we specified independent distributions for *H. pylori* prevalence among individuals with diabetes (prevalence range 40%–80%) and without diabetes (30%–60%), each within a specified range. Then, we established a prior probability distribution for the incidence of *H. pylori* gastric cancer. The 95% CI for the *H. pylori* and gastric cancer association was assigned between 2.3 and 3.7 based on a previous meta‐analysis.^26^

In order to have a general overview of past researches on the association between diabetes and gastric cancer, a systematic search using PubMed was conducted for articles on diabetes and gastric cancer risk published with keywords “gastric cancer” and “diabetes,” as well as “stomach cancer” and “diabetes.” We included published papers between 2000 and 2022 to maximize temporal relevance and to avoid greater heterogeneity in findings due to possible changes in diagnostic technology and improvements in treatment and management of both diabetes and gastric cancer over time (Figure S3). Selected studies were further screened via title, abstract, and full‐text review. Only the studies that assessed the risk of gastric cancer incidence due to diabetes and reported rate ratios were included for meta‐analysis and subgroup analysis. Random‐effect models were used to assess estimated effects, *I* ^2^ statistics to evaluate statistical heterogeneity among studies, and Egger's regression test and funnel plots to determine publication bias.

Estimates based on <10 cases were not reported. *p* values were two sided and considered statistically significant if <.05. Analyses were performed using SAS 9.4 (SAS Institute, Cary, NC, USA) and Stata 16.0 (Stata Corp, College Station, TX, USA).

## 3 RESULTS

A total of 8556 first primary incidence gastric cancer cases were identified (5676 men and 2880 women) during a median follow‐up of 14.7 years (77 866 634 person‐years; Table 1). At baseline, the median age of the participants was 52 years (range 15–103); they had a mean BMI of 23.1 kg/m^2^ (SD 3.4), 36% ever smoked, and 43% ever consumed alcohol. The overall prevalence of self‐reported diabetes was 4.8%.

**TABLE 1:** Baseline characteristics of participating cohorts for gastric cancer incidence analysis.

Country	Cohort name	Baseline survey years	Number of participants, N^a^	Follow‐up, median (range), years	Age at baseline, median (range), years	BMI, mean (SD), kg/m^2^	Male, %	Ever smoking, %	Ever drinking, %	Diabetes, %	Gastric cancer cases, N^b^
Japan	Aichi3P	1985	23 065	15.2 (0.0–15.5)	55 (40–103)	22.1 (2.9)	47.0	45.9	60.4	4.6	372
	JACC	1988–1990	74 462	19.4 (0.0–22.0)	57 (40–79)	22.8 (3.0)	42.2	35.3	46.4	5.4	1339
	JPHC I	1990–1992	42 136	22.6 (0.0–23.0)	50 (40–59)	23.6 (3.0)	48.2	40.3	50.1	4.0	1007
	JPHC II	1992–1995	55 317	19.7 (0.0–20.0)	54 (40–69)	23.5 (3.1)	47.7	40.0	49.1	5.6	1351
	LSS	1963–1993	41 960	21.9 (0.1–38.9)	52 (19–98)	22.0 (3.5)	45.5	48.2	45.1	7.2	2016
	Takayama	1992	30 781	15.6 (0.0–15.6)	56 (35–101)	22.2 (2.9)	46.2	22.2	77.2^c^	4.5	662
Korea	KMCC	1993–2005	18 759	14.2 (0.0–21.5)	54 (15–91)	23.6 (3.3)	40.1	36.6	41.6	4.6	439
	KNCC	2007–2015	36 484	9.2 (0.1–15.4)	50 (16–85)	23.9 (3.0)	51.5	45.1	67.5	2.4	305
China	SMHS	2001–2006	61 464	12.2 (0.0–15.0)	55 (40–75)	23.7 (3.1)	100	69.6	33.7	6.3	562
	SWHS	1996–2000	73 356	18.1 (0.0–20.0)	53 (40–71)	24.0 (3.4)	0	2.8	2.3	4.3	453
India	MCS	1991–1997	101 197	5.6 (0.0–11.9)	52 (35–98)	22.2 (4.1)	68.2	45.9	NA	4.5	50
Total			558 981	14.7 (0.0–38.9)	52 (15–103)	23.1 (3.4)	49.9	36.0	43.0	4.8	8556

Abbreviations: Aichi3P, Three‐Prefecture Cohort Study Aichi; BMI, body mass index; GC, gastric cancer; JACC, Japan Collaborative Cohort Study; JPHC, Japan Public Health Center‐Based Prospective Study; KMCC, Korea Multicenter Cancer Cohort Study; KNCC, Korea National Cancer Center Cohort Study; LSS, Life Span Study; MCS, Mumbai Cohort Study; NA, no information available; SMHS, Shanghai Men's Health Study; SWHS, Shanghai Women's Health Study; Takayama, Takayama Study; SD, standard deviation.

^a^ Includes only eligible participants for the current pooled analysis.

^b^ Included only first primary gastric cancer cases.

^c^ Current alcohol consumption variable was used for Takayama cohort.

**FIGURE 1:**
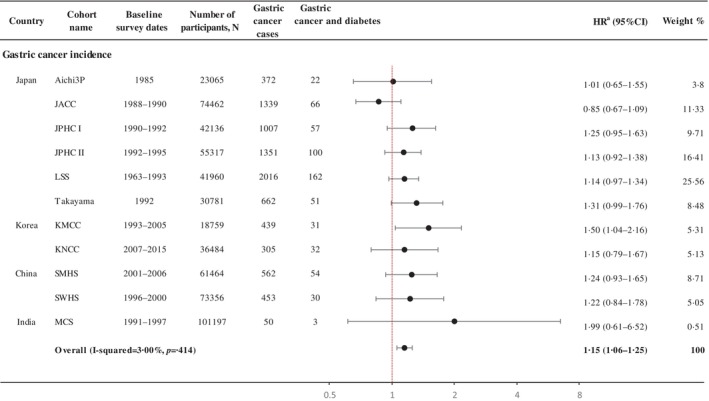
Association between diabetes and gastric cancer incidence. ^a^Adjusted for sex, smoking (ever/never), alcohol consumption (ever/never), education (low/high), and body mass index (kg/m^2^, continuous) depending on availability in each cohort. Aichi3P, Three‐Prefecture Cohort Study Aichi; CI, confidence interval; DM, diabetes mellitus; GC, gastric cancer; HR; hazard ratio; JACC, Japan Collaborative Cohort Study; JPHC, Japan Public Health Center‐Based Prospective Study; KMCC, Korea Multicenter Cancer Cohort Study; KNCC, Korea National Cancer Center Cohort Study; LSS, Life Span Study; MCS, Mumbai Cohort Study; SMHS, Shanghai Men's Health Study; SWHS, Shanghai Women's Health Study; Takayama, Takayama Study.

Individuals with a history of diabetes had a 15% increased risk of incident gastric cancer (HR 1.15, 95% CI 1.06–1.25; *I* ^2^ = 3.00%, *p* = .41; Figure 1). Among individual cohorts, KMCC showed a significant association (HR 1.50, 95% CI 1.04–2.16), and JACC showed a nonsignificant inverse association (HR 0.85, 95% CI 0.67–1.09).

Diabetes increased the risk of gastric cancer by 12% (HR 1.12, 95% CI 1.01–1.23; *I* ^2^ = 0.00%) in men and 31% in women (1.31, 1.07–1.60; *I* ^2^ = 30.00%, *p*_heterogeneity_ = .18; Table 2). By country, Korea showed the largest association for overall gastric cancer (HR 1.32, 95% CI 1.01–1.71), although the pattern was similar across the four countries (*p*_heterogeneity_ = .47). The risk was higher in those who enrolled after 1990 (1990–1999; HR 1.22, 95% CI 1.07–1.39; and 2000–2015; HR 1.23, 95% CI 1.00–1.51), born between 1930 and 1949 (HR 1.19, 95% CI 1.06–1.34), enrolled in their 50 (HR 1.20, 95% CI 1.04–1.39) and diagnosed with gastric cancer in their 60 (HR 1.28, 95% CI 1.10–1.49), although heterogeneity between these subgroups was not found (Table 2).

**TABLE 2:** Association between diabetes and gastric cancer incidence by demographic characteristics and lifestyle factors.

Subgroup analysis	No. of studies	No. participants	No. GC cases	No. GC and DM cases	Pooled HR^a^ (95% CI)	Heterogeneity within subgroups		Heterogeneity between subgroups^b^
						*I* ^2^, %	*p* value^c^	
Demographic characteristics								
Gender								
Male	10	279 096	5676	441	1.12 (1.01–1.23)	0.0	0.68	0.18
Female	10	279 885	2880	167	1.31 (1.07–1.60)	30.2	0.17	
Country								
Japan	6	267 721	6747	458	1.11 (0.99–1.25)	26.2	0.24	0.47
Korea	2	55 243	744	63	1.32 (1.01–1.71)	0.7	0.32	
China	2	134 820	1015	84	1.23 (0.98–1.55)	0.0	0.95	
India	1	101 197	50	3	1.99 (0.61–6.52)			
Enrollment year								
1963–1979	1	27 606	1638	116	1.17 (0.97–1.42)			0.32
1980–1989	3	90 701	1683	94	0.98 (0.80–1.21)	0.0	0.69	
1990–1999	8	329 467	4202	296	1.22 (1.07–1.39)	9.9	0.35	
2000–2015	4	111 186	1033	102	1.23 (1.00–1.51)	0.0	0.85	
Birth year								
<1930	10	121 379	4030	279	1.04 (0.86–1.26)	38.4	0.1	0.49
1930–1949	11	298 534	4005	309	1.19 (1.06–1.34)	0.0	0.95	
≥1950	5	139 068	521	20	1.11 (0.71–1.75)	0.0	0.91	
Age at enrollment (years)								
<50	9	230 000	1786	84	1.06 (0.84–1.33)	0.0	0.62	0.66
50–59	10	170 835	2884	282	1.20 (1.04–1.39)	0.0	0.83	
≥60	10	158 146	3886	608	1.14 (0.96–1.35)	48.1	0.04	
Age at cancer diagnosis (years)								
<60	10	146 470	1474	64	1.23 (0.92–1.66)	20.0	0.26	0.35
60–69	11	181 979	2420	185	1.28 (1.10–1.49)	0.0	0.85	
≥70	11	230 532	4662	359	1.09 (0.93–1.29)	42.1	0.07	

Abbreviations: CI, confidence interval; DM, diabetes mellitus; GC, gastric cancer; HR, hazard ratio.

^a^ Summary estimate based on random‐effects model. Adjusted for sex, smoking (ever/never), alcohol consumption (ever/never), education (low/high), and body mass index (kg/m^2^, continuous) depending on availability in each cohort.

^b^ *p* value for heterogeneity between subgroups.

^c^ *p* value for heterogeneity within each group.

**FIGURE 2:**
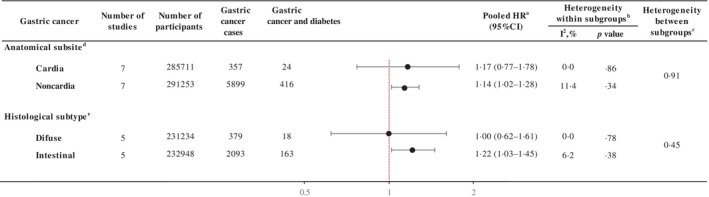
Association between diabetes and gastric cancer incidence by anatomical subsite and histological subtype in selected cohorts. ^a^Summary estimate based on random‐effects model. Adjusted for sex, smoking (ever/never), alcohol consumption (ever/never), education (low/high), and body mass index (kg/m^2^, continuous) depending on availability in each cohort. ^b^ *p* value for heterogeneity within each group. ^c^ *p* value for heterogeneity between subgroups. ^d^Cohorts included Three‐Prefecture Cohort Study Aichi, Japan Collaborative Cohort Study, Japan Public Health Center‐Based Prospective Study I, Japan Public Health Center‐Based Prospective Study II, Life Span Study, Korea Multicenter Cancer Cohort Study, Korea National Cancer Center Cohort Study. ^e^Cohorts included: Three‐Prefecture Cohort Study Aichi, Japan Collaborative Cohort Study, Japan Public Health Center‐Based Prospective Study I, Japan Public Health Center‐Based Prospective Study II, Life Span Study. CI, confidence interval; HR, hazard ratio.

Diabetes was significantly associated with the risk of noncardia (HR 1.14, 95% CI 1.02–1.28) but not with cardia gastric cancer (HR 1.17, 95% CI 0.77–1.78) with a nonsignificant heterogeneity between the subsites (*p*_heterogeneity_ = .91; Figure 2). A positive association was observed for the intestinal (HR 1.22, 95% CI 1.02–1.46) but not for the diffuse subtype (HR 1.00, 95% CI 0.62–1.61; *p*_heterogeneity_ = .45; Figure 2).

**FIGURE 3:**
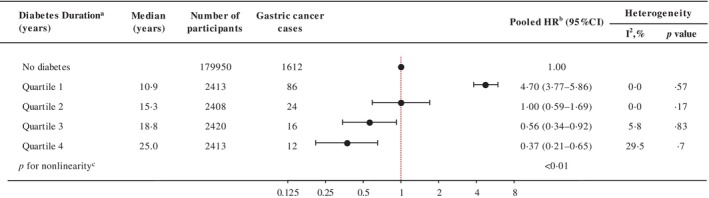
Association between diabetes duration at censoring (between age at diagnosis and age at last cohort follow‐up) gastric cancer incidence in selected cohorts. Selected cohorts: Korea Multicenter Cancer Cohort Study, Korea National Cancer Center Cohort Study, Shanghai Men's Health Study, Shanghai Women's Health Study. ^a^Diabetes duration was categorized by cohort‐specific quartiles. ^b^Summary estimate based on random‐effects model adjusted for sex, smoking (ever/never), alcohol consumption (ever/never), education (low/high), and body mass index (kg/m^2^, continuous) depending on availability in each cohort. ^c^Nonlinear trend *p* value based on dose–response random‐effects model. CI, confidence interval; HR, hazard ratio.

The risk of gastric cancer in participants with diabetes relative to people without diabetes (at cohort entry) increased during the first decade of diabetes (first quartile, median 10.9 years; HR 4.70, 95% CI 3.77–5.86) and then decreased, resulting in a nonlinear inverted V or lambda‐shaped curve (∧) (*p* < .01 for nonlinear relationship; Figure 3). When diabetes diagnosis was limited to diabetes duration assessed at baseline, the risk of gastric cancer increased by 40% in the second quartile (median = 2.7 years; HR 1.40, 95% CI 1.01–1.95) and by 39% in the third quartile (5.6 years; HR 1.39, 95% CI 1.00–1.93) compared to those without a diabetes diagnosis. Within 10 years of follow‐up, the risk of gastric cancer was linearly related to the duration of diabetes at baseline (*p* trend = .01; Table S3). When restricted to newly diagnosed diabetes, similarly, the risk of gastric cancer increased in the second quartile (median = 6.9 years; HR 7.20, 95% CI 4.76–10.89).

The analyses of the association between diabetes and gastric cancer mortality consisted of 580 663 participants and 5084 gastric cancer deaths during a median follow‐up of 14.7 years (Table S4). Diabetes was associated with a 15% increased risk of death from gastric cancer (HR 1.15, 95% CI 1.03–1.28; *I* ^2^ = 0.00%, *p* = .68; Table S5). Analyses by various subgroups did not show any differential associations (Table S6). Despite relatively few cases (*N* = 97), diabetes was positively associated with cardia (HR 2.18, 95% CI 1.14–4.14) but not with noncardia gastric cancer mortality (1.08, 0.95–1.24; *p*_heterogeneity_ = .04; Table S7). Gastric cancer mortality in participants with diabetes relative to participants without diabetes increased until ~10 years of diabetes duration but decreased afterwards (Table S8).

In the sensitivity analyses, although the overall association was attenuated when cases occurring within the first 2 years of follow‐up were excluded, the magnitude of association between diabetes and gastric cancer incidence (HR 1.11, 95% CI 1.00–1.24) and mortality (HR 1.11, 95% CI 0.96–1.29) remained similar (Table S9). The associations restricted to participants >30 years old at baseline did not change for gastric cancer incidence (HR 1.15, 95% CI 1.06–1.26) and mortality (HR 1.15, 95% CI 1.03–1.28; Table S9). In the competing risk analyses, association for gastric cancer incidence slightly decreased but remained significant (HR 1.12, 95% CI 1.03–1.22); whereas for mortality, the estimate was attenuated and nonsignificant (HR 1.10, 95% CI 0.99–1.23; Table S10). For the diabetes duration, the inverted lambda‐shaped curve remained consistent (*p*_nonlinear_ = .0; Table S11). In the leave‐one‐out analysis, none of the cohorts included in the study substantially differed (<4%) in both gastric incidence and mortality analyses (Table S12). The quantitative probabilistic bias analysis found a 0%–34% bias if *H. pylori* was unmeasured (Table S13).

A total of 26 studies regarding the association between diabetes and gastric cancer risk were included in the meta‐analysis (Table S14). Diabetes was associated with a higher risk of overall gastric cancer (risk ratio [RR] 1.24, 95% CI 1.15–1.34) with statistically significant heterogeneity across the studies (*I* ^2^ = 86.60%, *p* < .001; Table S15). Analyses by various subgroups did not show any differential associations by sex, region, study design, method of diabetes assessment, anatomical subsite, publication year, follow‐up time, and adjustment variables (Table S16). The leave‐one‐out analysis revealed <1% differences by each excluded study (Table S17). The funnel plot demonstrated a symmetrical distribution (Figure S4) with no evidence of publication bias was detected by Egger's regression tests (*p* ≥ .05).

## 4 DISCUSSION

In this pooled analysis of Asian populations, we found that diabetes was associated with a higher risk of both gastric cancer incidence and mortality. To our knowledge, this is the largest study to comprehensively assess the association between diabetes duration and gastric cancer in the Asian population.

Several mechanisms have been proposed for the link between diabetes and gastric cancer. First, insulin and hyperglycemia may directly promote gastric cancer by overstimulating tumorigenic pathways such as PI3K/AKT/mTOR,^27^ insulin‐like growth factor‐1 (IGF‐1), strongly linked to insulin resistance, promotes gastric cancer cell proliferation and invasion via the Ras/RAF/MEK/ERK and JAK1/STAT3 cellular signaling pathways.^28^ Hyperglycemia also enhances gastric carcinoma proliferation and migration via thr Pin1/BRD4 transcriptional pathway, involved in the hyperglycemia‐induced inflammatory process.^29^ Second, *H. pylori* infection, a known risk factor for gastric cancer, is maintained in hyperglycemia, allowing the bacteria to grow and survive longer in patients with diabetes.^30^ An in vitro study reported enhanced adhesion, growth, and viability of the *H. pylori* cytotoxin‐associated gene A antigen, an oncoprotein, under higher glucose concentrations.^31^ Finally, some glucose‐lowering therapies, such as sulfonylureas and insulin therapy, may increase gastric cancer risk by interacting with insulin and insulin IGF‐1R signaling.^32^ In contrast, metformin, which counteracts insulin resistance, can reduce the risk by activating AMPK and inhibiting IGF‐1R, respectively.^33^

Our observation of the positive association between diabetes and gastric cancer risk corroborates previous studies.^4^ Our up‐to‐date meta‐analysis, including subgroup analysis by sex, anatomical subsite, type of study, and average follow‐up time, confirmed the positive direction with gastric cancer. Compared to previous meta‐analyses,^4^, ^6^ our meta‐analysis exclusively focused on studies published after January 2000 to ensure temporal relevance. Furthermore, we conducted a subgroup analysis focusing on specific anatomical subsites (cardia and noncardia gastric cancer), addressing a knowledge gap in previous meta‐analyses. Additionally, we extended the follow‐up period in the subgroup analysis by categorizing studies into <10 years and ≥10 years of follow‐up. This allowed for a better understanding of the effects of diabetes on gastric cancer, considering the long onset time of the disease. Although the heterogeneity among studies was high in ours and other meta‐analyses,^4^, ^6^ mainly due to study quality and effect measures,^4^ the heterogeneity among Asian cohorts included in our analysis was relatively low.

We observed that the magnitude of association was higher in women compared to men, although the difference was not statistically significant, which is also shown in our meta‐analysis. Although we cannot dismiss a possible interaction between women's hormonal condition and gastric cancer risk, our meta‐analysis supports our findings that diabetes is associated with increased risk of gastric cancer in both men and women.

Our study found no significant difference in the association between diabetes and gastric cancer incidence between cardia and noncardia subsites. Although the results could be affected by the relatively small number of cardia gastric cancer cases, there was a slightly higher positive association between cardia gastric cancer and diabetes. When combined with published literature, diabetes was significantly associated with cardia gastric cancer. *H. pylori* infection, known to be more associated with noncardia gastric cancers, may interact with diabetes as described here; whereas, obesity, more reportedly linked to cardia cancers, may in tandem work with the effects of diabetes.^34^ Similarly, despite the absence of statistical difference between the two histological subsites, only intestinal gastric cancer showed a positive association. Although both histological subsites may be associated with *H. pylori* infection, the link is stronger with intestinal subtypes, which again may synergize with diabetes in the carcinogenesis.^35^ Further research is warranted in disentangling the effects of *H. pylori* infection and obesity in the diabetes‐gastric cancer subtypes association.

Diabetes and gastric cancer mortality association were previously investigated within the Asian Cohort Consortium as part of an analysis for all‐cause and cause‐specific mortality.^36^, ^37^ With a longer follow‐up (12.7 vs 14.7 years), we reevaluated the association between diabetes and gastric cancer mortality overall and newly by subgroups and anatomical subsites. Although we found a positive association between diabetes and gastric cancer mortality, the association disappeared in the 2‐year lag time analysis and competing risk analysis. Therefore, diabetes may not be a main risk for gastric cancer mortality, as previous studies have suggested.^36^, ^37^

We found that within 10 year‐term of diabetes duration increases the risk of gastric cancer, supporting the evidence on the potential role of hyperglycemia‐hyperinsulinemia in gastric cancer development. We also observed that after 11 years of diabetes duration, the association seems to weaken or even reverse. Our results were similar to a previous prospective study of two cohorts from the United States, which reported that overall cancer risk gradually reduced after 8 years of diabetes duration.^38^ Although this study did not include gastric cancer as an outcome, the pattern was similarly shown in colorectal, breast, endometrial, and prostate cancers.^38^ This may imply a harvesting effect, whereby there may be an increase in cancer detection among diabetes patients. Alternatively, hyperinsulinemia in early diabetes may play a more significant role than hyperglycemia in promoting cancer development because, after a particular diabetes stage, the progressive depletion of beta cell function leads to a steady decline in endogenous insulin production.^38^

The major strength of our study includes a large sample size of Asian populations with a long‐term follow‐up, which gave us the statistical power to conduct stratified and sensitivity analyses. We also performed lag analyses to minimize recall bias and reverse causality, and competing risk analyses to mitigate survival bias. Furthermore, we had comprehensive data from each cohort at the individual level for covariate adjustments and heterogeneity assessment, avoiding some limitations of meta‐analyses based on published results. Additionally, all study participants were selected from the general population, ensuring generalizability in the targeted population. The number of exclusions due to missing data on self‐reported diabetes or any other covariates was not particularly large, <13% and 5%, respectively. Furthermore, we had information on gastric cancer diagnosis and mortality by anatomical subsite and histological subtype. All cases were validated by national cancer registries or by active, long‐term follow‐up.

Our study has some limitations. First, we lacked information about *H. pylori* infection. However, despite the synergistic effect of hyperglycemia and *H. pylori* infection on gastric cancer, there is evidence that diabetes increases the risk of gastric cancer even after *H. pylori* eradication,^11^ implying different and independent mechanisms on gastric cancer carcinogenesis. Moreover, our quantitative probabilistic bias analysis revealed a relative wide range bias, which may be explained by regional differences in *H. pylori* prevalence. Second, diabetes diagnosis was self‐reported at recruitment, which may have introduced misclassification bias because some participants may have been unaware of their diabetes diagnosis or developed it during the cohort follow‐up. However, misclassification should not have occurred differentially between cases and noncases.^19^ Also, the subgroup analysis in our meta‐analysis also showed that the association of self‐reported diabetes and gastric cancer risk did not differ from the medical record‐confirmed results. Diabetes type was unavailable; nonetheless, we can assume that type 2 diabetes predominated, as only 0.43% of diabetes cases were diagnosed before age 30, and our findings were unaffected by the exclusion of patients below this threshold. Third, we could not control for other factors related to gastric cancer; therefore, we could not rule out residual confounding. However, we have adjusted our models for known gastric cancer factors such as obesity, alcohol consumption, smoking, and education as a proxy of income to minimize confounding bias. Although we have a large number of cancer cases, some subgroup analyses may have insufficient statistical power. We cannot rule out the possibility of selection bias, particularly in histology analysis, given the limited percentage of cases with this information (50%); nevertheless, the anatomical subsite accounts for ~65%–100% of cases. Finally, we lacked information on the type of diabetes treatment and hypoglycemic drug use. Even with this information, assessing the influence of diabetes treatment on gastric cancer is challenging due to significant heterogeneity in the effects of different hypoglycemic drugs on gastric cancer risk.^32^, ^33^ Additional long‐term prospective cohort studies with detailed information on diabetes treatment and hypoglycemic drugs are warranted to clarify the associations between diabetes/diabetes duration and gastric cancer risk.

In conclusion, we found that diabetes was associated with an increased gastric cancer incidence. The largest gastric cancer risk was found within the first 10 years after a diabetes diagnosis. As diabetes is highly prevalent in Asia, our findings underline the importance of lifestyle and metabolic health maintenance in the relatively healthy general population, and proposes a closer surveillance in the early stages of diabetes for gastric cancer prevention.

## AUTHOR CONTRIBUTIONS

Katherine De la Torre and Minkyo Song are joint first authors. Katherine De la Torre contributed to design, methodology and study visualization; led the data analysis and interpretation of the results; and drafted the first draft of the manuscript. Minkyo Song conceptualized the design of the study, methodology, and visualization; supervised data analysis; interpreted the findings; and created the first draft. Sarah Krull Abe and Eiko Saito contributed as project managers. Md. Shafiur Rahman and Md. Rashedul Islam performed data curation. Sukhong Min contributed to meta‐analysis and drafted some sections of the original manuscript. Dan Huang verified data onsite and contributed to data analysis. Yu Chen provided critical revision of the manuscript, and clinical input. Prakash C. Gupta, Norie Sawada, Akiko Tamakoshi, Xiao‐Ou Shu, Wanqing Wen, Ritsu Sakata, Jeongseon Kim, Chisato Nagata, Hidemi Ito, Sue K. Park, Myung‐Hee Shin, Mangesh S. Pednekar, Shoichiro Tsugane, Takashi Kimura, Yu‐Tang Gao, Hui Cai, Keiko Wada, Isao Oze, Aesun Shin, and Yoon‐Ok Ahn obtained funding, provided resources, verified data analysis, and provided critical revision of the manuscript. Habibul Ahsan, Paolo Boffetta, Kee Seng Chia, Keitaro Matsuo, You‐Lin Qiao, Nathaniel Rothman, Wei Zheng, and Manami Inoue contributed to project supervision and provided critical revision of the manuscript. Daehee Kang obtained funding, contributed to the study conceptualization and design, supervision, and interpretation of results and commented on the paper draft. All authors contributed to the critical revision of the manuscript, and approved the final version. Daehee Kang, Minkyo Song, and Katherine De la Torre act as the guarantors of the study and accept full responsibility for the work and/or the conduct of the study, had access to the data, and controlled the decision to publish.

## FUNDING INFORMATION

This work was supported by the following grants for study design, data collection and participant recruitment: Mumbai Cohort Study funded by the International Agency for Research on Cancer, Lyon, France; Clinical Trials Service Unit, Oxford, UK; World Health Organization, Geneva, Switzerland; Japan Collaborative Cohort Study (JACC), the National Cancer Center Research and Development Fund, grant‐in‐Aid for Cancer Research; Grant for Health Services and grant for Comprehensive Research on Cardiovascular and Life‐Style Related Diseases from the Ministry of Health, Labour and Welfare, Japan; and grant for the Scientific Research from the Ministry of Education, Culture, Sports, Science and Technology, Japan; Shanghai Women's Health Study (SWHS), the US National Cancer Institute (NCI) (grant numbers R37 CA070867 and UM1 CA182910; Principal investigator: W. Zheng); Shanghai Men's Health Study (SMHS), the US NCI (grant number UM1 CA173640; Principal investigator: X.O. Shu); Japan Public Health Center‐based prospective Study (JPHC Study) I and II, National Cancer Center Research and Development Fund [23‐A‐31 (toku) and 26‐A‐2; since 2011] and a Grant‐in‐Aid for Cancer Research from the Ministry of Health, Labour and Welfare of Japan (from 1989 to 2010; Principal investigator: S. Tsugane); Life Span Study Cohort, the Japanese Ministry of Health, Labour and Welfare and the US Department of Energy; Korean National Cancer Screenee Cohort Study, National Cancer Center National Cancer Center Korea Research Grant (grant number 2210990, 24H1080; Principal investigator: J. Kim); 3 Prefecture Aichi Study, The Japanese Ministry of the Environment (formerly, Environment Agency; Principal investigator: K. Matsuo); Takayama Study, National Cancer Center Research and Development Fund (Principal investigator: C. Nagata); Korean Multicenter Cancer Cohort (KMCC), the National Research Foundation of Korea (NRF) grant funded by the Korea government (MSIP; No. NRF‐2016R1A2B4014552; Principal investigator: S.K. Park; Seoul Male Cancer Cohort, the National R&D Program for Cancer Control, National R&D Program for Cancer Control, Ministry of Health & Welfare, Republic of Korea (0520160‐1; Principal Investigator: Myung‐Hee Shin); National Cancer Center Japan Research and Development Fund (30‐A‐15,23‐A‐31(toku),26‐A‐2,29‐A‐4; Principal investigator: M. Inoue).

## CONFLICT OF INTEREST STATEMENT

The authors declare that they have no known competing financial interests or personal relationships that could have appeared to influence the work reported in this paper.

## Supporting information


**TABLE S1.** Characteristics of cohorts in the current study of Asia Cohort Consortium.
**FIGURE S1.** A flow chart of study participants for gastric cancer incidence analysis.
**FIGURE S2.** A flow chart of study participants for gastric cancer mortality analysis.
**TABLE S2.** Diabetes‐related deaths included in the competing risk analysis.
**TABLE S3.** Association between diabetes duration and gastric cancer incidence in selected cohorts.
**TABLE S4.** Baseline characteristics of participating cohorts for mortality analysis.
**TABLE S5.** Association between diabetes and gastric cancer mortality.
**TABLE S6.** Association between diabetes and gastric cancer mortality by demographic characteristics and lifestyle factors.
**TABLE S7.** Association between diabetes and gastric cancer mortality overall and by anatomical subsite in selected cohorts.
**TABLE S8.** Association between diabetes duration and gastric cancer mortality in selected cohorts.
**TABLE S9.** Association of diabetes and gastric cancer incidence and mortality, restricted to 2‐year lag time and participants older than age 30 years at recruitment.
**TABLE S10.** Competing risk analysis of the association between diabetes and gastric cancer incidence and mortality.
**TABLE S11.** Competing risk analysis of the association between diabetes duration and gastric cancer incidence and mortality in selected cohorts.
**TABLE S12.** A sensitivity analysis using leave‐one‐out method for the association between diabetes and gastric cancer incidence and mortality in the Asia Cohort Consortium.
**TABLE S13.** Probabilistic sensitivity analysis for unmeasured confounding (*H. pylori*) in the association between diabetes and gastric cancer.
**TABLE S14.** Characteristics of studies for meta‐analysis on diabetes and gastric cancer association.
**FIGURE S3.** Literature search and selection process for the meta‐analysis studies on diabetes and gastric cancer incidence.
**TABLE S15.** A meta‐analysis of studies on diabetes and gastric cancer incidence.
**TABLE S16.** A subgroup analysis for the meta‐analysis of studies on diabetes and gastric cancer association.
**TABLE S17.** A sensitivity analysis using leave‐one‐out method for the meta‐analysis of studies on diabetes and gastric cancer association.
**FIGURE S4.** A funnel plot for studies on diabetes and gastric cancer incidence.

## References

[1] Sung H , Ferlay J , Siegel RL , et al. Global cancer statistics 2020: GLOBOCAN estimates of incidence and mortality worldwide for 36 cancers in 185 countries. CA Cancer J Clin. 2021;71(3):209‐249. 33538338 10.3322/caac.21660

[2] Nanditha A , Ma RC , Ramachandran A , et al. Diabetes in Asia and the Pacific: implications for the global epidemic. Diabetes Care. 2016;39(3):472‐485. 26908931 10.2337/dc15-1536

[3] Ling S , Brown K , Miksza JK , et al. Risk of cancer incidence and mortality associated with diabetes: a systematic review with trend analysis of 203 cohorts. Nutr Metab Cardiovasc Dis. 2021;31(1):14‐22. 33223399 10.1016/j.numecd.2020.09.023

[4] Guo J , Liu C , Pan J , Yang J . Relationship between diabetes and risk of gastric cancer: a systematic review and meta‐analysis of cohort studies. Diabetes Res Clin Pract. 2022;187:109866. 35398143 10.1016/j.diabres.2022.109866

[5] Bae JM . Diabetes history and gastric cancer risk: different results by types of follow‐up studies. Asian Pac J Cancer Prev. 2022;23(5):1523‐1528. 35633534 10.31557/APJCP.2022.23.5.1523 PMC9587861

[6] Miao ZF , Xu H , Xu YY , et al. Diabetes mellitus and the risk of gastric cancer: a meta‐analysis of cohort studies. Oncotarget. 2017;8(27):44881‐44892. 28415651 10.18632/oncotarget.16487 PMC5546528

[7] Dabo B , Pelucchi C , Rota M , et al. The association between diabetes and gastric cancer: results from the stomach cancer pooling project consortium. Eur J Cancer Prev. 2022;31(3):260‐269. 34183534 10.1097/CEJ.0000000000000703 PMC8709871

[8] Kim SK , Jang JY , Kim DL , et al. Site‐specific cancer risk in patients with type 2 diabetes: a nationwide population‐based cohort study in Korea. Korean J Intern Med. 2020;35(3):641‐651. 32392663 10.3904/kjim.2017.402 PMC7214364

[9] Kuriki K , Hirose K , Tajima K . Diabetes and cancer risk for all and specific sites among Japanese men and women. Eur J Cancer Prev. 2007;16(1):83‐89. 17220709 10.1097/01.cej.0000228404.37858.40

[10] Inoue M , Iwasaki M , Otani T , Sasazuki S , Noda M , Tsugane S . Diabetes mellitus and the risk of cancer: results from a large‐scale population‐based cohort study in Japan. Arch Intern Med. 2006;166(17):1871‐1877. 17000944 10.1001/archinte.166.17.1871

[11] Cheung KS , Chan EW , Chen L , Seto WK , Wong ICK , Leung WK . Diabetes increases risk of gastric cancer after *Helicobacter pylori* eradication: a territory‐wide study with propensity score analysis. Diabetes Care. 2019;42(9):1769‐1775. 31296646 10.2337/dc19-0437

[12] Yang HJ , Kang D , Chang Y , et al. Diabetes mellitus is associated with an increased risk of gastric cancer: a cohort study. Gastric Cancer. 2020;23(3):382‐390. 31853749 10.1007/s10120-019-01033-8

[13] Chiu M , Austin PC , Manuel DG , Shah BR , Tu JV . Deriving ethnic‐specific BMI cutoff points for assessing diabetes risk. Diabetes Care. 2011;34(8):1741‐1748. 21680722 10.2337/dc10-2300 PMC3142051

[14] Narayan KMV , Kanaya AM . Why are south Asians prone to type 2 diabetes? A hypothesis based on underexplored pathways. Diabetologia. 2020;63(6):1103‐1109. 32236731 10.1007/s00125-020-05132-5 PMC7531132

[15] Song M , Rolland B , Potter JD , Kang D . Asia cohort consortium: challenges for collaborative research. J Epidemiol. 2012;22(4):287‐290. 22672913 10.2188/jea.JE20120024 PMC3798645

[16] Zheng W , McLerran DF , Rolland B , et al. Association between body‐mass index and risk of death in more than 1 million Asians. N Engl J Med. 2011;364(8):719‐729. 21345101 10.1056/NEJMoa1010679 PMC4008249

[17] Rolland B , Smith BR , Potter JD . Coordinating centers in cancer epidemiology research: the Asia cohort consortium coordinating center. Cancer Epidemiol Biomarkers Prev. 2011;20(10):2115‐2119. 21803842 10.1158/1055-9965.EPI-11-0391 PMC3189300

[18] Von Elm E , Altman DG , Egger M , Pocock SJ , Gøtzsche PC , Vandenbroucke JP . The strengthening the reporting of observational studies in epidemiology (STROBE) statement: guidelines for reporting observational studies. Lancet. 2007;370(9596):1453‐1457. 18064739 10.1016/S0140-6736(07)61602-X

[19] Boffetta P , McLerran D , Chen Y , et al. Body mass index and diabetes in Asia: a cross‐sectional pooled analysis of 900,000 individuals in the Asia cohort consortium. PLoS ONE. 2011;6(6):e19930. 21731609 10.1371/journal.pone.0019930 PMC3120751

[20] Orsini N , Li R , Wolk A , Khudyakov P , Spiegelman D . Meta‐analysis for linear and nonlinear dose‐response relations: examples, an evaluation of approximations, and software. Am J Epidemiol. 2012;175(1):66‐73. 22135359 10.1093/aje/kwr265 PMC3244608

[21] Kohl M , Plischke M , Leffondre K , Heinze G . PSHREG: a SAS macro for proportional and nonproportional subdistribution hazards regression. Comput Methods Programs Biomed. 2015;118(2):218‐233. 25572709 10.1016/j.cmpb.2014.11.009 PMC4673318

[22] Orsini N , Bellocco R , Bottai M , Wolk A , Greenland S . A tool for deterministic and probabilistic sensitivity analysis of epidemiologic studies. Stata J. 2008;8(1):29‐48.

[23] Bener A , Agan AF , Al‐Hamaq A , Barisik CC , Ozturk M , Omer A . Prevalence of *Helicobacter pylori* infection among type 2 diabetes mellitus. Adv Biomed Res. 2020;9:27. 33072639 10.4103/abr.abr_248_19 PMC7532836

[24] Kouitcheu Mabeku LB , Noundjeu Ngamga ML , Leundji H . *Helicobacter pylori* infection, a risk factor for type 2 diabetes mellitus: a hospital‐based cross‐sectional study among dyspeptic patients in Douala‐Cameroon. Sci Rep. 2020;10(1):12141. 32699242 10.1038/s41598-020-69208-3 PMC7376106

[25] Vafaeimanesh J , Parham M , Bagherzadeh M . *Helicobacter pylori* infection prevalence: is it different in diabetics and nondiabetics? Indian J Endocrinol Metab. 2015;19(3):364‐368. 25932391 10.4103/2230-8210.152773 PMC4366774

[26] Helicobacter, Cancer Collaborative G . Gastric cancer and *Helicobacter pylori*: a combined analysis of 12 case control studies nested within prospective cohorts. Gut. 2001;49(3):347‐353. 11511555 10.1136/gut.49.3.347 PMC1728434

[27] Saisana M , Griffin SM , May FEB . Insulin and the insulin receptor collaborate to promote human gastric cancer. Gastric Cancer. 2022;25(1):107‐123. 34554347 10.1007/s10120-021-01236-y PMC8732810

[28] Su C , Wang W , Wang C . IGF‐1‐induced MMP‐11 expression promotes the proliferation and invasion of gastric cancer cells through the JAK1/STAT3 signaling pathway. Oncol Lett. 2018;15:7000‐7006. 29731870 10.3892/ol.2018.8234 PMC5921070

[29] Yu J , Hu D , Wang L , et al. Hyperglycemia induces gastric carcinoma proliferation and migration via the Pin1/BRD4 pathway. Cell Death Discovery. 2022;8(1):224. 35461311 10.1038/s41420-022-01030-4 PMC9035156

[30] Zhou X , Zhang C , Wu J , Zhang G . Association between *Helicobacter pylori* infection and diabetes mellitus: a meta‐analysis of observational studies. Diabetes Res Clin Pract. 2013;99(2):200‐208. 23395214 10.1016/j.diabres.2012.11.012

[31] Sheu S‐M , Cheng H , Kao C‐Y , Yang Y‐J , Wu J‐J , Sheu B‐S . Higher glucose level can enhance the *H. pylori* adhesion and virulence related with type IV secretion system in AGS cells. J Biomed Sci. 2014;21(1):96. 25296847 10.1186/s12929-014-0096-9 PMC4196111

[32] Dulskas A , Patasius A , Kaceniene A , Linkeviciute‐Ulinskiene D , Zabuliene L , Smailyte G . A cohort study of Antihyperglycemic medication exposure and gastric cancer risk. J Clin Med. 2020;9(2):435. 32033451 10.3390/jcm9020435 PMC7073990

[33] Zhang J , Wen L , Zhou Q , He K , Teng L . Preventative and therapeutic effects of metformin in gastric cancer: a new contribution of an old friend. Cancer Manag Res. 2020;12:8545‐8554. 32982447 10.2147/CMAR.S264032 PMC7505710

[34] Kamangar F , Dawsey SM , Blaser MJ , et al. Opposing risks of gastric cardia and noncardia gastric adenocarcinomas associated with *Helicobacter pylori* seropositivity. J Natl Cancer Inst. 2006;98(20):1445‐1452. 17047193 10.1093/jnci/djj393

[35] Uemura N , Okamoto S , Yamamoto S , et al. *Helicobacter pylori* infection and the development of gastric cancer. N Engl J Med. 2001;345(11):784‐789. 11556297 10.1056/NEJMoa001999

[36] Chen Y , Wu F , Saito E , et al. Association between type 2 diabetes and risk of cancer mortality: a pooled analysis of over 771,000 individuals in the Asia cohort consortium. Diabetologia. 2017;60(6):1022‐1032. 28265721 10.1007/s00125-017-4229-z PMC5632944

[37] Yang JJ , Yu D , Wen W , et al. Association of diabetes with all‐cause and cause‐specific mortality in Asia: a pooled analysis of more than 1 million participants. JAMA Netw Open. 2019;2(4):e192696. 31002328 10.1001/jamanetworkopen.2019.2696 PMC6481439

[38] Hu Y , Zhang X , Ma Y , et al. Incident type 2 diabetes duration and cancer risk: a prospective study in two US cohorts. JNCI. 2021;113(4):381‐389. 33225344 10.1093/jnci/djaa141 PMC8599903

