# Supplementary material for: Diabetes and gastric cancer incidence and mortality in the Asia Cohort Consortium: A pooled analysis of more than a half million participants
Source: J Diabetes. 2024 May 16;16(6):e13561. doi: 10.1111/1753-0407.13561 (PMC11096812; doi:10.1111/1753-0407.13561)
Supplement: Supplementary file 1 — TABLE S1. Characteristics of cohorts in the current study of Asia Cohort Consortium. FIGURE S1. A flow chart of study participants for gastric cancer incidence analysis. FIGURE S2. A flow chart of study participants for gastric cancer mortality analysis. TABLE S2. Diabetes‐related deaths included in the competing risk analysis. TABLE S3. Association between diabetes duration and gastric cancer incidence in selected cohorts. TABLE S4. Baseline characteristics of participating cohorts for mortality analysis. TABLE S5. Association between diabetes and gastric cancer mortality. TABLE S6. Association between diabetes and gastric cancer mortality by demographic characteristics and lifestyle factors. TABLE S7. Association between diabetes and gastric cancer mortality overall and by anatomical subsite in selected cohorts. TABLE S8. Association between diabetes duration and gastric cancer mortality in selected cohorts. TABLE S9. Association of diabetes and gastric cancer incidence and mortality, restricted to 2‐year lag time and participants older than age 30 years at recruitment. TABLE S10. Competing risk analysis of the association between diabetes and gastric cancer incidence and mortality. TABLE S11. Competing risk analysis of the association between diabetes duration and gastric cancer incidence and mortality in selected cohorts. TABLE S12. A sensitivity analysis using leave‐one‐out method for the association between diabetes and gastric cancer incidence and mortality in the Asia Cohort Consortium. TABLE S13. Probabilistic sensitivity analysis for unmeasured confounding (H. pylori) in the association between diabetes and gastric cancer. TABLE S14. Characteristics of studies for meta‐analysis on diabetes and gastric cancer association. FIGURE S3. Literature search and selection process for the meta‐analysis studies on diabetes and gastric cancer incidence. TABLE S15. A meta‐analysis of studies on diabetes and gastric cancer incidence. TABLE S16. A subgroup analysis for t [file JDB-16-e13561-s001.docx]

**Supplementary Materials**

[**Supplementary Table 1.** Characteristics of cohorts in the current study of Asia Cohort Consortium 2](#_Toc148978730)

[**Supplementary Figure 1.** A flow chart of study participants for gastric cancer incidence analysis 4](#_Toc148978731)

[**Supplementary Figure 2.** A flow chart of study participants for gastric cancer mortality analysis 5](#_Toc148978732)

[**Supplementary Table 2.** Diabetes-related deaths included in the competing risk analysis 6](#_Toc148978733)

[**Supplementary Table 3.** Association between diabetes duration and gastric cancer incidence in selected cohorts 7](#_Toc148978734)

[**Supplementary Table 4**. Baseline characteristics of participating cohorts for mortality analysis 8](#_Toc148978735)

[**Supplementary Table 5.** Association between diabetes and gastric cancer mortality 9](#_Toc148978736)

[**Supplementary Table 6.** Association between diabetes and gastric cancer mortality by demographic characteristics and lifestyle factors 10](#_Toc148978737)

[**Supplementary Table 7.** Association between diabetes and gastric cancer mortality overall and by anatomical subsite in selected cohorts 11](#_Toc148978738)

[**Supplementary Table 8.** Association between diabetes duration and gastric cancer mortality in selected cohorts 12](#_Toc148978739)

[**Supplementary Table 9.** Association of diabetes and gastric cancer incidence and mortality, restricted to two-years lag time and participants older than age 30 years at recruitment 13](#_Toc148978740)

[**Supplementary Table 10.** Competing risk analysis of the association between diabetes and gastric cancer incidence and mortality 14](#_Toc148978741)

[**Supplementary Table 11.** Competing risk analysis of the association between diabetes duration and gastric cancer incidence and mortality in selected cohorts. 15](#_Toc148978742)

[**Supplementary Table 12.** A sensitivity analysis using leave-one-out method for the association between diabetes and gastric cancer incidence and mortality in the Asia Cohort Consortium 16](#_Toc148978743)

[**Supplementary Table 13.** Probabilistic sensitivity analysis for unmeasured confounding (H. pylori) in the association between Diabetes and Gastric Cancer 17](#_Toc148978744)

[**Supplementary Table 14.** Characteristics of studies for meta-analysis on diabetes and gastric cancer association 18](#_Toc148978745)

[**Supplementary Figure 3.** Literature search and selection process for the meta-analysis studies on diabetes and gastric cancer incidence 21](#_Toc148978746)

[**Supplementary Table 15.** A meta-analysis of studies on diabetes and gastric cancer incidence 22](#_Toc148978747)

[**Supplementary Table 16.** A subgroup analysis for the meta-analysis of studies on diabetes and gastric cancer association 23](#_Toc148978748)

[**Supplementary Table 17.** A sensitivity analysis using leave-one-out method for the meta-analysis of studies on diabetes and gastric cancer association 24](#_Toc148978749)

[**Supplementary Figure 4.** A funnel plot for studies on diabetes and gastric cancer incidence 25](#_Toc148978750)

[**Supplementary References** 26](#_Toc148978751)

# **Supplementary Table 1.** Characteristics of cohorts in the current study of Asia Cohort Consortium

| **Cohort name** | **Study Location, total participant number, and characteristics** | **Questionnaire assessment** | **Diabetes assessment** | **Outcome assessment** | **Age/year at diabetes diagnosis** | **Gastric cancer-related information (% of total cases)** | **Covariates included in models** |
| --- | --- | --- | --- | --- | --- | --- | --- |
| Three-Prefecture Cohort Study Aichi (Aichi3P)^1^ | N=33538 aged over 40 residents of Nagoya and Inuyama City of Aichi prefecture, Japan | Self-administered | “Has a doctor ever told you that you have any of the following diseases? – diabetes (current/past/never)” | Death and cancer incidence information were retrieved from residence certificates, vital statistics, and the local cancer registry. |  | Anatomical subsite (cardia 4.6%, non-cardia 58.6%) and histological subtype (intestinal 10.75%, luminal 5.65%) | Sex, smoking (ever/never), alcohol consumption (ever/never), and body mass index (continuous) |
| Japan Collaborative Cohort Study for Evaluation of Cancer Risk (JACC)^2,3^ | N=110585 (46395 men and 64190 women) aged 40 to 79 years residents in Hokkaido, Tohoku, Kanto, Chubu, Kinkim Chugoku, and Kyushu districts, Japan | Self-administered | “Have you had any of these physician-diagnosed diseases? – diabetes (no/yes‐under medication/yes‐cured/yes‐not under medication) | Cancer incidence and causes of death were collected through population-based registers or local hospital records |  | Anatomical subsite (cardia 3.36%, non-cardia 77.82%) and histological subtype (intestinal 23.60%, luminal 2.09%) | Sex, smoking (ever/never), alcohol consumption (ever/never), education level (low/high), and body mass index (continuous) |
| Japan Public Health Center-Based Prospective Study I (JPHC I)^4^ | N=61595 aged 40 to 59 residents of Iwate, Akita, Tokyo, Nagano, and Okinawa, Japan | Self-administered | “Have you ever been diagnosed with any of the following diseases? – diabetes (yes/no)” | Participants in the study were followed up for the occurrence of cancer and death using data from cancer and residential registries |  | Anatomical subsite (cardia 6.16%, non-cardia 86.49%) and histological subtype (intestinal 41.71%, luminal 7.05%) | Sex, smoking (ever/never), alcohol consumption (ever/never), education level (low/high), and body mass index (continuous) |
| Japan Public Health Center-Based Prospective Study II (JPHC II)^4^ | N=78825 aged 40 to 69 residents of Ibaraki, Niigata, Osaka, Kochi, Nagasaki, and Okinawa, Japan | Self-administered | “Have you ever been diagnosed with any of the following diseases? – diabetes (yes/no)” | Same as JPHC1 |  | Anatomical subsite (cardia 6.07%, non-cardia 88.38%) and histological subtype (intestinal 37.3%, luminal 7.6%) | Sex, smoking (ever/never), alcohol consumption (ever/never), and body mass index (continuous) |
| Korea Multicenter Cancer Cohort Study (KMCC)^5^ | N=19688 cancer-free women and men over 18 years old residents?in Haman, Choongju, Uljin, and Pohang, Republic of Korea | Interviewer-administered | “Have you ever been diagnosed by a doctor at a hospital for any of the diseases? – no/yes-cured/yes-under medication” | Cancer incidence and cause of death information were retrieved from the central and regional cancer registries, health insurance medical records databases, and national death certificates | Year at diabetes diagnosis | Anatomical subsite (cardia 3.42%, non-cardia 88.61%) | Sex, smoking (ever/never), alcohol consumption (ever/never), education level (low/high), and body mass index (continuous) |
| Korea National Cancer Center Cohort Study (KNCC)^6^ | N=16304 men and women over 30 years of age, residents in Haman, Sancheong, Changwon, Chungju, Chuncheon, Republic of Korea | Interviewer-administered | “Have you ever been diagnosed by a doctor at a hospital for any of the diseases? – no/yes-cured/yes-under medication” | Information on cancer incidence and cause of death was retrieved annually through the Korea National Cancer Incidence Database of the Korean Central Cancer Registry and the Cause of Death Database of Statistics | Year at diabetes diagnosis | Anatomical subsite (cardia 5.25%, non-cardia 94.75%) | Sex, smoking (ever/never), alcohol consumption (ever/never), education level (low/high), and body mass index (continuous) |
| Mumbai Cohort Study (MCS)^7^ | N=148173 aged over 35 years in Mumbai, India | Interviewer-administered | “Do/did you suffer from any major disease in the last five years? – diabetes (yes/no) | Cancer incidence and cause of death were ascertained by data linkage to local cancer and death registry databases and vital statistics |  |  | Sex, smoking (ever/never), education level (low/high), body mass index (continuous) |
| Life Span Study (LSS)^8^ | N=120321 atomic bomb survivors (82214 from Hiroshima and 38107 from Nagasaki) We included only those exposed to less than 0.01 grays of bomb radiation. | Self-administered | “Have you ever had any of the following diseases? – diabetes (yes/no)” | The cohort is linked to Japan's official family registry system for death information. Cancer incidence is linked to the local cancer registries in Hiroshima and Nagasaki and the Japan National Cancer Registry |  | Anatomical subsite (cardia 5.95%, non-cardia 94.05%) and histological subtype (intestinal 40.33%, luminal 7.74%) | Sex, smoking (ever/never), alcohol consumption (ever/never), education level (low/high), and body mass index (continuous) |
| Seoul Male Cohort (SeoulM)^9^ | N=14533 male participants aged 40 to 59 years residing in Seoul Province, Republic of Korea | Self-administered | “Have you ever been diagnosed with any of the following diseases? – diabetes (yes/no/I do not know)” | Death information was retrieved from the National Statistics Office | Age at diabetes diagnosis. | Anatomical subsite (cardia 4.04%, non-cardia 95.96%) | Smoking (ever/never), alcohol consumption (ever/never), education level (low/high), body mass index (continuous). |
| Shanghai Men’s Health Study (SMHS)^10^ | N=61480 cancer-free men aged 40 to 74 in urban Shanghai communities, China | Self-administered | “Have you ever been diagnosed with any of the following diseases? – diabetes (yes/no) | Record linkages to the Shanghai Cancer Registry (SCR), Shanghai Vital Statistics Registry, the Shanghai Residential Registry, and the Changning District Health Information System | Age at diabetes first diagnosis |  | smoking (ever/never), alcohol consumption (ever/never), education level (low/high), body mass index (continuous) |
| Shanghai Women’s Health Study (SWHS)^11^ | N=74942 women aged 40 to 70 years in urban Shanghai communities, China | Self-administered | “Have you ever been diagnosed with any of the following diseases? – diabetes (yes/no) | Study participants were followed by an in-person assessment every two years and were routinely linked to cancer and vital statistic registries | Age at diabetes first diagnosis |  | smoking (ever/never), alcohol consumption (ever/never), education level (low/high), body mass index (continuous) |
| Takayama Study (Takayama)^12^ | N=31552 residents (14427 men and 17125 women) aged 35 years or older in Takayama city, Gifu Prefecture, Japan | Self-administered | Has a doctor ever told you that you have any of the following diseases? – diabetes (current/past/never) | Information on cancer diagnosis, death, or emigration was obtained from the cancer registry, death certificates, and basic resident or family registration databases |  |  | sex, smoking (ever/never), alcohol current consumption (yes/no), education (low/high), body mass index (continuous) |


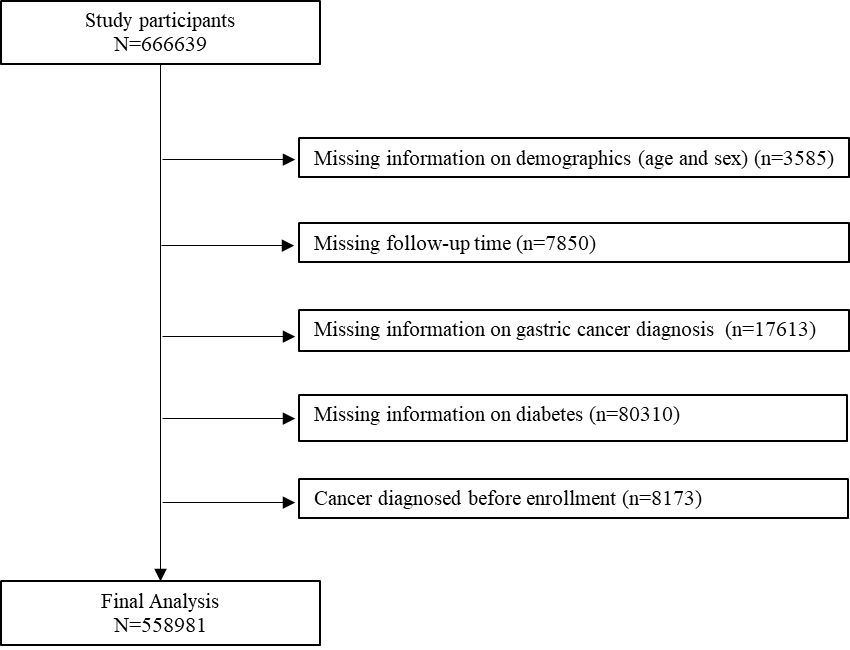


* The numbers presented in each box for exclusion are not exclusive

# **Supplementary Figure 1.** A flow chart of study participants for gastric cancer incidence analysis


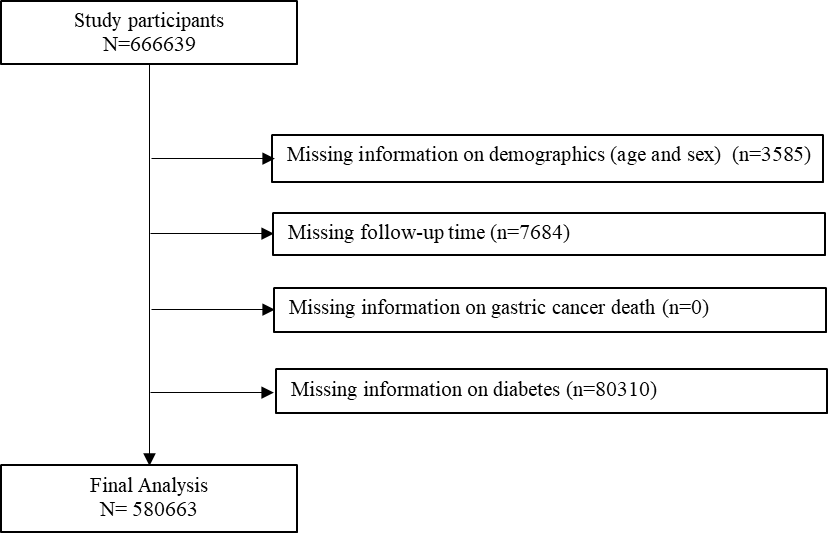


* The numbers presented in each box for exclusion are not exclusive

# **Supplementary Figure 2.** A flow chart of study participants for gastric cancer mortality analysis

# **Supplementary Table 2.** Diabetes-related deaths included in the competing risk analysis

|  | **International Classification of Diseases Ninth Revision (ICD-9)** | **International Classification of Diseases Tenth Revision (ICD-10)** |
| --- | --- | --- |
| Cerebrovascular diseases | **433** Occlusion and stenosis of precerebral arteries  **434** Cerebral thrombosis  **436** cerebrovascular disease | **I63** Cerebral infarction  **I64** Stroke, not specified as haemorrhage or infarction |
| Ischemic heart disease | **410** Acute myocardial infarction  **411** Other acute and subacute forms of ischemic heart disease | **I21** Acute myocardial infarction |
| Hypertensive disease | **401** Essential hypertension  **402** Hypertensive heart disease  **403** Hypertensive renal disease  **404** Hypertensive heart and renal disease | **I10** Essential (primary) hypertension  **I11** Hypertensive heart disease  **I12** Hypertensive renal disease  **I13** Hypertensive heart and renal disease |
| Chronic Kidney disease | **584-586,588-589** Disorders resulting from impaired renal function, and small kidney of unknown cause | **N18** Chronic kidney disease  Incl.: chronic renal failure |
| Diabetes | **250** Diabetes mellitus | **E11** Type 2 diabetes mellitus |

# **Supplementary Table 3.** Association between diabetes duration and gastric cancer incidence in selected cohorts

|  | **Diabetes Duration^a^ (years)** | **Median  (years)** | **Number of Participants** | **Gastric Cancer Cases** | **Pooled HR^b^ (95%CI)** | **Heterogeneity** | |
| --- | --- | --- | --- | --- | --- | --- | --- |
|  |  |  |  |  |  | **I^2^,%** | **p-value** |
|  | **Diabetes duration at baseline (between age at diabetes diagnosis and age at cohort recruitment/baseline)** | | | | | | |
|  | No diabetes |  | 179950 | 1612 | 1.00 |  |  |
|  | Quartile 1 | 0.8 | 2294 | 26 | 1.14 (0.77-1.68) | 0.00 | 0.62 |
|  | Quartile 2 | 2.7 | 2325 | 36 | 1.40 (1.01-1.95) | 0.00 | 0.86 |
|  | Quartile 3 | 5.6 | 2544 | 40 | 1.39 (1.00-1.93) | 5.80 | 0.36 |
|  | Quartile 4 | 12.0 | 2491 | 36 | 1.28 (0.86-1.92) | 29.50 | 0.77 |
|  | P- trend^c^ |  |  |  | 0.01 |  |  |
|  | **Restricted to newly diagnosed diabetes (diabetes duration <1 year at recruitment)** | | | | | | |
|  | No diabetes |  | 179950 | 1,612 | 1.00 |  |  |
|  | Quartile 1 | 6.9 | 447 | 23 | 7.20 (4.76-10.89) | 0.00 | 0.85 |
|  | Quartile 2 | 12.0 | 451 | NA | NA | NA | NA |
|  | Quartile 3 | 13.4 | 453 | NA | NA | NA | NA |
|  | Quartile 4 | 17.4 | 449 | NA | NA | NA | NA |
|  | P- trend^c^ |  |  |  | NA |  |  |

Selected cohorts: Korea Multicenter Cancer Cohort Study, Korea National Cancer Center Cohort Study; Shanghai Men’s Health Study, Shanghai Women’s Health Study.

Quartiles are study-specific

Abbreviation: NA, not applicable due to small number of cases

^a^ Diabetes duration was categorized by cohort-specific quartiles

^b^ Summary estimate based on random-effects model

^c^ Linear trend and non-linear p-values are based on dose-response random-effects model

# **Supplementary Table 4**. Baseline characteristics of participating cohorts for mortality analysis

| **Country** | **Cohort Name** | **Baseline Survey Dates** | **Number of Participants, N^a^** | **Follow-up, Median (Range),  Years** | **Age at Baseline, Median (Range),  Years** | **BMI,  Mean (SD), kg/m^2^** | **Male,  %** | **Ever Smoking,  %** | **Ever Drinking,  %** | **Diabetes,  %** | **Gastric Cancer Deaths,  N^b^** |
| --- | --- | --- | --- | --- | --- | --- | --- | --- | --- | --- | --- |
| Japan |  |  |  |  |  |  |  |  |  |  |  |
|  | Aichi3P | 1985 | 23111 | 15.2 (0.0-15.5) | 55 (40-103) | 22.1 (2.9) | 47.9 | 45.8 | 60.4 | 4.7 | 246 |
|  | JACC | 1988-1990 | 75376 | 19.4 (0.0-22.0) | 57 (40-79) | 22.8 (3.0) | 42.0 | 35.2 | 46.4 | 5.5 | 1088 |
|  | JPHC I | 1990-1992 | 43051 | 22.6 (0.0-23.0) | 50 (40-59) | 23.6 (3.0) | 47.9 | 40.1 | 49.8 | 4.0 | 419 |
|  | JPHC II | 1992-1995 | 56535 | 19.7 (0.0-20.0) | 54 (40-69) | 23.5 (3.1) | 47.4 | 39.8 | 48.8 | 5.7 | 654 |
|  | RERF | 1963-1993 | 41960 | 21.9 (0.1-38.9) | 52 (19-98) | 22.0 (3.5) | 45.5 | 48.2 | 45.1 | 7.2 | 1267 |
|  | Takayama | 1992 | 31552 | 15.6 (0.0-15.6) | 56 (35-101) | 22.2 (2.9) | 45.6 | 7.1 | 76.9^b^ | 4.6 | 325 |
| Korea |  |  |  |  |  |  |  |  |  |  |  |
|  | KMCC | 1993-2005 | 19106 | 14.2 (0.0-21.5) | 54 (15-91) | 23.6 (3.3) | 40.0 | 36.6 | 41.5 | 4.7 | 185 |
|  | KNCC | 2007-2015 | 38541 | 9.2 (0.1-15.4) | 50 (16-85) | 23.9 (3.0) | 50.9 | 44.6 | 66.7 | 6.2 | 20 |
|  | SeoulM | 1992-1993 | 13833 | 9.2 (0.1-15.4) | 49 (16-85) | 23.4 (2.4) | 100.0 | 76.1 | 72.9 | 2.9 | 99 |
| China |  |  |  |  |  |  |  |  |  |  |  |
|  | SMHS | 2001-2006 | 61464 | 12.2 (0.0-15.0) | 55 (40-75) | 23.7 (3.1) | 100.0 | 69.6 | 33.7 | 6.3 | 410 |
|  | SWHS | 1996-2000 | 74937 | 18.1 (0.0-20.0) | 53 (40-71) | 24.0 (3.4) | 0.0 | 2.8 | 2.2 | 4.3 | 401 |
| India |  |  |  |  |  |  |  |  |  |  |  |
|  | MCS | 1991-1997 | 101197 | 5.6 (0.0-11.9) | 52 (35-98) | 22.2 (4.1) | 68.2 | 22.3 | NA | 4.5 | 50 |
| Total |  |  | 580663 | 14.7 (0.0-38.9) | 53 (15-103) | 23.1 (3.4) | 50.8 | 36.8 | 43.8 | 4.8 | 5084 |

Aichi3P, Three-Prefecture Cohort Study Aichi; JACC, Japan Collaborative Cohort Study; JPHC, Japan Public Health Center-Based Prospective Study; KMCC, Korea Multicenter Cancer Cohort Study; KNCC, Korea National Cancer Center Cohort Study; MCS, Mumbai Cohort Study; SeoulM; Seoul Male Cohort SMHS, Shanghai Men’s Health Study; SWHS, Shanghai Women’s Health Study; Takayama, Takayama Study; yrs, years; BMI, body mass index; SD, standard deviation; GC gastric cancer; NA, no information available

a Includes only eligible participants for the current pooled analysis

b Current alcohol consumption variable was used for Takayama cohort

# **Supplementary Table 5.** Association between diabetes and gastric cancer mortality

| Aichi3P, Three-Prefecture Cohort Study Aichi; JACC, Japan Collaborative Cohort Study; JPHC, Japan Public Health Center-Based Prospective Study; KMCC, Korea Multicenter Cancer Cohort Study; KNCC, Korea National Cancer Center Cohort Study; LSS, Life Span Study; MCS, Mumbai Cohort Study; SeoulM; Seoul Male Cohort SMHS, Shanghai Men’s Health Study; SWHS, Shanghai Women’s Health Study; Takayama, Takayama Study; GC, gastric cancer; DM, diabetes mellitus; HR; hazard ratio. | |
| --- | --- |
| ^a^ Adjusted for sex, smoking (ever/never), alcohol consumption (ever/never), education (low/high) and body mass index (kg/m^2^, continuous) depending on availability in each cohort |  |

# **Supplementary Table 6.** Association between diabetes and gastric cancer mortality by demographic characteristics and lifestyle factors

| **Subgroup Analysis** | **No. of studies** | **No. participants** | **No. GC deaths** | **No. GC & DM cases** | **Pooled HR^a^ (95%CI)** | **Heterogeneity within subgroups** | | **Heterogeneity between subgroups^c^** |
| --- | --- | --- | --- | --- | --- | --- | --- | --- |
|  |  |  |  |  |  | **I^2^,%** | **p-value^b^** |  |
| **Demographics characteristics** | | |  |  |  |  |  |  |
| **Sex** |  |  |  |  |  |  |  |  |
| Male | 11 | 295116 | 3384 | 255 | 1.12 (0.99-1.27) | 0.0 | 0.54 | 0.27 |
| Female | 10 | 285547 | 1700 | 358 | 1.30 (1.03-1.65) | 18.2 | 0.28 |  |
| **Country** |  |  |  |  |  |  |  |  |
| Japan | 6 | 271585 | 3919 | 272 | 1.14 (1.00-1.30) | 8.4 | 0.36 | 0.81 |
| Korea | 3 | 71480 | 304 | 14 | 1.06 (0.62-1.82) | 0.0 | 0.77 |  |
| China | 2 | 136401 | 811 | 69 | 1.20 (0.90-1.59) | 23.5 | 0.25 |  |
| India | 1 | 101197 | 50 | 3 | 1.98 (0.60-6.48) | . | . |  |
| **Enroll year** |  |  |  |  |  |  |  |  |
| 1963-1979 | 1 | 27606 | 1060 | 75 | 1.19 (0.94-1.50) | . | . | 0.81 |
| 1980-1989 | 4 | 91418 | 1191 | 80 | 1.13 (0.90-1.42) | 0.0 | 0.79 |  |
| 1990-1999 | 9 | 348090 | 2339 | 162 | 1.21 (1.00-1.47) | 23.9 | 0.23 |  |
| 2000-2015 | 4 | 113549 | 494 | 41 | 1.01 (0.73-1.40) | 0.0 | 0.89 |  |
| **Birth year** |  |  |  |  |  |  |  |  |
| <1930 | 11 | 137051 | 2764 | 196 | 1.11 (0.96-1.29) | 0.0 | 0.74 | 0.67 |
| 1930-1949 | 11 | 302656 | 2098 | 155 | 1.19 (1.01-1.41) | 0.0 | 0.73 |  |
| ≥1950 | 8 | 140956 | 222 | 7 | 1.48 (0.69-3.18) | 0.0 | 0.87 |  |
| **Age at enroll (years)** | |  |  |  |  |  |  |  |
| <50 | 12 | 239349 | 900 | 40 | 1.20 (0.87-1.66) | 0.0 | 0.95 | 0.94 |
| 50-59 | 12 | 179790 | 1522 | 97 | 1.18 (0.96-1.45) | 0.0 | 0.63 |  |
| ≥60 | 12 | 161524 | 1662 | 221 | 1.14 (0.98-1.32) | 11.9 | 0.33 |  |
| **Age at cancer death (years)** | |  |  |  |  |  |  |  |
| <60 | 11 | 150707 | 855 | 42 | 1.62 (1.19-2.21) | 0.0 | 0.83 | 0.10 |
| 60-69 | 12 | 191959 | 1229 | 77 | 1.13 (0.91-1.40) | 0.0 | 0.98 |  |
| ≥70 | 12 | 237997 | 3000 | 239 | 1.13 (0.98-1.29) | 0.0 | 0.52 |  |

^a^ Summary estimate based on random-effects model. Adjusted for sex, smoking (ever/never), alcohol consumption (ever/never), education (low/high), body mass index (kg/m2, continuous) depending on availability in each cohort

^b^ P value for heterogeneity within each group

^c^ P value for heterogeneity between subgroups

# **Supplementary Table 7.** Association between diabetes and gastric cancer mortality overall and by anatomical subsite in selected cohorts

Selected cohorts: Three-Prefecture Cohort Study Aichi, Japan Collaborative Cohort Study, Japan Public Health Center-Based Prospective Study I, Japan Public Health Center-Based Prospective Study II, Life Span Study, Korea Multicenter Cancer Cohort Study, Korea National Cancer Center Cohort Study

^a^ Summary estimate based on random-effects model. Adjusted for sex, smoking (ever/never), alcohol consumption (ever/never), education (low/high), body mass index (kg/m2, continuous) depending on availability in each cohort

^b^ P value for heterogeneity within each group

^c^ P value for heterogeneity between subgroups

# **Supplementary Table 8.** Association between diabetes duration and gastric cancer mortality in selected cohorts

|  | **Diabetes Duration^a^ (years)** | **Median  (years)** | **Number of Participants** | **Gastric Cancer Deaths** | **Pooled HR^b^ (95%CI)** | **Heterogeneity** | |
| --- | --- | --- | --- | --- | --- | --- | --- |
|  |  |  |  |  |  | **I^2^,%** | **p-value** |
|  | **Diabetes duration at censoring (between age at diabetes diagnosis and age at last cohort follow-up)** | | | | | | |
|  | No diabetes |  | 197047 | 1032 | 1.00 |  |  |
|  | Quartile 1 | 10.9 | 2519 | 51 | 5.39 (3.32-8.78) | 56.50 | 0.08 |
|  | Quartile 2 | 15.5 | 2644 | 11 | 0.77 (0.37-1.57) | 21.50 | 0.28 |
|  | Quartile 3 | 18.9 | 2588 | 10 | 0.56 (0.30-1.04) | 0.00 | 0.94 |
|  | Quartile 4 | 25.1 | 2598 | 11 | 0.60 (0.33-1.09) | 0.00 | 0.66 |
|  | P- for non linearity^c^ |  |  |  | <0.01 |  |  |
|  | **Diabetes duration at baseline (between age at diabetes diagnosis and age at cohort recruitment/baseline)** | | | | | | |
|  | No diabetes |  | 197047 | 1032 | 1.00 |  |  |
|  | Quartile 1 | 0.7 | 2474 | 15 | 0.99 (0.60-1.66) | 0.00 | 0.85 |
|  | Quartile 2 | 2.8 | 2669 | 21 | 1.23 (0.80-1.90) | 0.00 | 0.69 |
|  | Quartile 3 | 5.8 | 2575 | 24 | 1.49 (0.99-2.23) | 0.00 | 0.62 |
|  | Quartile 4 | 12.2 | 2631 | 23 | 1.30 (0.86-1.96) | 0.00 | 0.41 |
|  | P- trend^c^ |  |  |  | 0.04 |  |  |

Selected cohorts: Korea Multicenter Cancer Cohort Study, Korea National Cancer Center Cohort Study, Seoul Male Cancer Cohort, Shanghai Men’s Health Study, Shanghai Women’s Health Study

Results for the analysis restricted to newly diagnosed diabetes (diabetes duration <1 year at recruitment) were not included (estimates based on fewer than 10 cases)

^a^ Diabetes duration was categorized by cohort-specific quartiles

^b^ Summary estimate based on random-effects model

^c^ Linear trend and non-linear p-values are based on dose-response random-effects

# **Supplementary Table 9.** Association of diabetes and gastric cancer incidence and mortality, restricted to two-years lag time and participants older than age 30 years at recruitment

|  | **Gastric Cancer Incidence** | | | | |  | **Gastric Cancer Mortality** | | | | |
| --- | --- | --- | --- | --- | --- | --- | --- | --- | --- | --- | --- |
|  | **Overall** |  | **Male** |  | **Female** |  | **Overall** |  | **Male** |  | **Female** |
| **Two-years lag time** |  |  |  |  |  |  |  |  |  |  |  |
| Participants | 558161 |  | 278824 |  | 279724 |  | 580062 |  | 294690 |  | 285372 |
| Cohorts | 11 |  | 10 |  | 10 |  | 12 |  | 11 |  | 10 |
| Gastric cancer cases/deaths | 7736 |  | 5130 |  | 2606 |  | 4483 |  | 2958 |  | 1525 |
| Diabetes in gastric cancer cases | 538 |  | 390 |  | 148 |  | 313 |  | 221 |  | 92 |
| HR (95%CI)^a^ | 1.11 (1.00-1.24) |  | 1.07 (0.97 - 1.19) |  | 1.26 (1.01 - 1.59) |  | 1.11 (0.96-1.29) |  | 1.08 (0.87 - 1.30) |  | 1.27 (0.99 - 1.63) |
| **Participants older than 30 years at recruitment** | | | |  |  |  |  |  |  |  |  |
| Participants | 555677 |  | 278173 |  | 277504 |  | 577351 |  | 294191 |  | 283160 |
| Cohorts | 11 |  | 10 |  | 10 |  | 12 |  | 11 |  | 10 |
| Gastric cancer cases/deaths | 8544 |  | 5672 |  | 2872 |  | 5075 |  | 3381 |  | 1694 |
| Diabetes in gastric cancer cases | 608 |  | 441 |  | 167 |  | 358 |  | 255 |  | 103 |
| HR (95%CI)^a^ | 1.15 (1.06 - 1.26) |  | 1.12 (1.01-1.23) |  | 1.31 (1.07- 1.61) |  | 1.15 (1.03 - 1.28) |  | 1.12 (0.99-1.28) |  | 1.30 (1.03-1.65) |

^a^ Summary estimate based on random-effects model. Adjusted for sex, smoking (ever/never), alcohol consumption (ever/never), education (low/high), body mass index (kg/m^2^, continuous) depending on availability in each cohort

# **Supplementary Table 10.** Competing risk analysis of the association between diabetes and gastric cancer incidence and mortality

|  | **Gastric Cancer Incidence** | |  | **Gastric Cancer Mortality** | |
| --- | --- | --- | --- | --- | --- |
|  | **HR** | **95%CI** |  | **HR** | **95%CI** |
| Aichi3P | 0.95 | 0.62-1.47 |  | 1.09 | 0.66-1.79 |
| JACC | 0.84 | 0.65-1.08 |  | 1.05 | 0.81-1.35 |
| JPHC I | 1.22 | 0.93-1.60 |  | 1.07 | 0.69-1.67 |
| JPHC II | 1.11 | 0.90-1.36 |  | 0.87 | 0.63-1.20 |
| LSS | 1.12 | 0.97-1.30 |  | 1.15 | 0.94-1.42 |
| Takayama | 1.28 | 0.95-1.71 |  | 1.5 | 1.03-2.19 |
| KMCC | 1.44 | 1.00-2.08 |  | 1.1 | 0.58-2.09 |
| KNCC | 1.15 | 0.79-1.66 |  | 0.55 | 0.07-4.04 |
| SeoulM | NA | NA |  | 0.95 | 0.29-3.05 |
| SMHS | 1.18 | 0.89-1.57 |  | 0.97 | 0.69-1.38 |
| SWHS | 1.14 | 0.78-1.67 |  | 1.28 | 0.89-1.84 |
| MCS | 1.99 | 0.60-6.65 |  | 1.98 | 0.59-6.60 |
| Overall | 1.12 | 1.03-1.22 |  | 1.10 | 0.99-1.23 |

**Supplementary Table 11.** Competing risk analysis of the association between diabetes duration and gastric cancer incidence and mortality in selected cohorts.

|  |  | **Incidence (N=558981)** | | |  | **Mortality (N=580663)** | | |
| --- | --- | --- | --- | --- | --- | --- | --- | --- |
|  | **Diabetes Duration^a^ (years)** | **Pooled HR^b^ (95%CI)** | **Heterogeneity** | |  | **Pooled HR^b^ (95%CI)** | **Heterogeneity** | |
|  |  |  | **I^2^,%** | **p-value** |  |  | **I^2^,%** | **p-value** |
|  | **Diabetes duration at censoring** | |  |  |  |  |  |  |
|  | No diabetes | 1.00 |  |  |  | 1.00 |  |  |
|  | Quartile 1 | 3.93 (3.14-4.91) | 0.00 | 0.76 |  | 4.08 (2.51-6.63) | 54.20 | 0.09 |
|  | Quartile 2 | 0.98 (0.58-1.66) | 32.00 | 0.22 |  | 0.75 (0.36-1.54) | 22.50 | 0.28 |
|  | Quartile 3 | 0.56 (0.34-0.91) | 0.00 | 0.56 |  | 0.54 (0.29-1.01 | 0.00 | 0.94 |
|  | Quartile 4 | 0.36 (0.21-0.64) | 0.00 | 0.97 |  | 0.58 (0.32-1.05) | 0.00 | 0.66 |
|  | P- for non-linearity^c^ | <0.01 |  |  |  | <0.01 |  |  |
|  | **Diabetes duration at Baseline** | |  |  |  |  |  |  |
|  | No diabetes | 1.00 |  |  |  | 1.00 |  |  |
|  | Quartile 1 | 1.10 (0.74-1.63) | 0.00 | 0.67 |  | 0.96 (0.57-1.60) | 0.00 | 0.86 |
|  | Quartile 2 | 1.34 (0.96-1.87) | 0.00 | 0.87 |  | 1.15 (0.75-1.78) | 0.00 | 0.65 |
|  | Quartile 3 | 1.35 (0.96-1.88) | 9.10 | 0.35 |  | 1.39 (0.92-2.09) | 0.00 | 0.62 |
|  | Quartile 4 | 1.20 (0.81-1.77) | 26.20 | 0.26 |  | 1.16 (0.76-1.75) | 0.00 | 0.43 |
|  | P- trend^c^ | 0.04 |  |  |  | 0.18 |  |  |
|  | **Restricted to newly DM diagnosed within their baseline enrollment (DM duration <1 year at recruitment)** | | | | | | | |
|  | No diabetes | 1.00 |  |  |  |  |  |  |
|  | Quartile 1 | 5.79 (3.76-8.91) | 0.00 | 0.91 |  |  |  |  |
|  | Quartile 2 | 0.85 (0.12-6.11) | NA | NA |  |  |  |  |
|  | Quartile 3 | 1.56 (0.49-5.00) | NA | NA |  |  |  |  |
|  | Quartile 4 | NA | NA | NA |  |  |  |  |

Selected cohorts: Korea Multicenter Cancer Cohort Study, Korea National Cancer Center Cohort Study; Shanghai Men’s Health Study, Shanghai Women’s Health Study.

Abbreviation: NA, not applicable due to the small number of cases

^a^ Diabetes duration was categorized by cohort-specific quartiles

^b^ Summary estimate based on random-effects model

^c^ Linear trend and non-linear p-values are based on dose-response random-effects model

# **Supplementary Table 12.** A sensitivity analysis using leave-one-out method for the association between diabetes and gastric cancer incidence and mortality in the Asia Cohort Consortium

|  | **Gastric Cancer Incidence** | | |  | **Gastric Cancer Mortality** | | |
| --- | --- | --- | --- | --- | --- | --- | --- |
|  | **HR** | **95%CI** | **% difference^a^** |  | **HR** | **95%CI** | **% difference^a^** |
| All cohorts | 1.15 | 1.06-1.25 |  |  | 1.15 | 1.03-1.28 |  |
| No Aichi3P | 1.16 | 1.06-1.27 | 0.87 |  | 1.15 | 1.03-1.28 | 0.00 |
| No JACC | 1.19 | 1.09-1.30 | 3.48 |  | 1.17 | 1.04-1.32 | 1.74 |
| No JPHC I | 1.14 | 1.04-1.26 | 0.87 |  | 1.15 | 1.03-1.29 | 0.00 |
| No JPHC II | 1.16 | 1.05-1.28 | 0.87 |  | 1.19 | 1.06-1.33 | 3.48 |
| No LSS | 1.16 | 1.04-1.29 | 0.87 |  | 1.13 | 1.00-1.29 | -1.74 |
| No Takayama | 1.14 | 1.04-1.25 | 0.87 |  | 1.12 | 1.00-1.25 | -2.61 |
| No KMCC | 1.13 | 1.04-1.23 | -1.74 |  | 1.15 | 1.03-1.28 | 0.00 |
| No KNCC | 1.15 | 1.05-1.27 | 0.00 |  | 1.15 | 1.03-1.28 | 0.00 |
| No SeoulM | NA | NA | NA |  | 1.15 | 1.03-1.28 | 0.00 |
| No SMHS | 1.15 | 1.04-1.26 | 0.00 |  | 1.16 | 1.04-1.30 | 0.87 |
| No SWHS | 1.15 | 1.05-1.26 | 0.00 |  | 1.13 | 1.01-1.26 | -1.74 |
| No MCS | 1.15 | 1.05-1.25 | 0.00 |  | 1.14 | 1.03-1.28 | -0.87 |

Aichi3P, Three-Prefecture Cohort Study Aichi; JACC, Japan Collaborative Cohort Study; JPHC, Japan Public Health Center-Based Prospective Study; KMCC, Korea Multicenter Cancer Cohort Study; KNCC, Korea National Cancer Center Cohort Study; MCS, Mumbai Cohort Study; SeoulM; Seoul Male Cohort SMHS, Shanghai Men’s Health Study; SWHS, Shanghai Women’s Health Study; Takayama, Takayama Study.

NA, no information available

^a^ % difference between HR of analysis excluding the indicated cohort vs. all cohort analysis

# **Supplementary Table 13.** Probabilistic sensitivity analysis for unmeasured confounding (H. pylori) in the association between Diabetes and Gastric Cancer

| **Assumed independent prevalence ranges** | | **Conventional Analysis** |  | **Systematic error** |  | **Systematic and random error** | **Bias %** |
| --- | --- | --- | --- | --- | --- | --- | --- |
| ***H. pylori* in Diabetes** | ***H. pylori* non-Diabetes** | **OR 95%CI** |  | **OR 95%CI** |  | **OR 95%CI** |  |
| 0.4-0.7 | 0.4-0.7 | 1.64 (1.50-1.78) |  | 1.64 (1.16-2.31) |  | 1.64 (1.15-2.34) | 0.00 |
| 0.5-0.8 | 0.4-0.7 | 1.64 (1.50-1.78) |  | 1.43 (1.03-1.96) |  | 1.43 (1.02-1.99) | 14.69 |
| 0.5-0.8 | 0.4-0.6 | 1.64 (1.50-1.78) |  | 1.33 (1.01-1.75) |  | 1.33 (0.99-1.77) | 23.31 |
| 0.5-0.8 | 0.3-0.6 | 1.64 (1.50-1.78) |  | 1.22 (0.84-1.72) |  | 1.2 (0.83-1.73) | 34.43 |
| 0.5-0.7 | 0.4-0.6 | 1.64 (1.50-1.78) |  | 1.42 (1.12-1.77) |  | 1.42 (1.10-1.80) | 15.49 |
| 0.5-0.7 | 0.3-0.6 | 1.64 (1.50-1.78) |  | 1.31 (0.92-1.75) |  | 1.30 (0.92-1.77) | 7.69 |

# **Supplementary Table 14.** Characteristics of studies for meta-analysis on diabetes and gastric cancer association

| **Author (Year)** | **Country** | **Study Design, Mean Follow Up Time if Cohort** | **Number of Participants and Characteristics** | **Diabetes Assessment** | **Outcome Assessment** | **Gastric Cancer Cases, N** | **Effect Estimate (95%CI)** | **Adjustment Variables** |
| --- | --- | --- | --- | --- | --- | --- | --- | --- |
| Rousseau (2006)^13^ | Canada | Case-control | N=3616 males aged 35-70 years | Self-report | Pathology | 226 | Male  OR 0.9 (0.4-1.9) | Age, family income, years of schooling, ethnicity, proxy status, BMI, smoking, beta-carotene consumption, alcohol consumption |
| Khan (2006)^14^ | Japan | Cohort  8.1 yrs | N=56881 (23378 males, 33503 females) aged 40-79 years | Self-report | Cancer registry | 631 | Male RR 0.72 (0.40-1.09)  Female RR 0.26 (0.08-0.82) | Age, BMI, smoking, drinking |
| Rapp (2006)^15^ | Austria | Cohort  8.4 yrs | N=140813 (63585 males, 77228 females) with ages between 19-95 years | Fasting blood glucose | Cancer registry | 219 | Male HR 0.84 (0.38-1.87) | Age, smoking, occupation, BMI |
| Inoue (2006)^16^ | Japan | Cohort  10.7 yrs | N=97771 (46548 males, 51223 females) 40-69 years | Self-report | Hospital records, cancer registry and death records | 1339 | Male HR 1.09 (0.79-1.50)  Female HR 1.92 (1.06-3.47) | Age, study area, history of cerebrovascular disease and ischemic heart disease, smoking, alcohol, BMI, leisure-time physical activity, green vegetable intake, coffee intake |
| Kuriki (2007)^17^ | Japan | Case-control | N=59440 (19540 males, 39900 females) aged 40-80 years | Self-report | Cancer registry | 1950 | Male OR 1.16 (0.93-1.44)  Female OR 1.70 (1.16-2.48) | Age, BMI, alcohol, smoking, exercise, bowel movement, family history of cancer and diabetes, dietary restriction, raw vegetable intake, greasy food intake, snacking |
| Ogunleye (2009)^18^ | Scotland | Cohort  4.0 yrs | N=28731 (15314 males, 13,417 females) without age limit | Hospital records | Cancer registry | 62 | Overall  RR 0.77 (0.36-1.66) | Deprivation deciles, matched for age, sex and clinic |
| Chodick (2010)^19^ | Israel | Cohort  8.0 yrs | N=100595 (52913 males and 47682 females) aged 21 years or more | Diabetes registry | Cancer registry | 307 | Male HR 1.44 (0.98-2.11)  Female HR 0.99 (0.55-1.80) | Age, region, socioeconomic status, use of healthcare year prior to index date, BMI, and history of cardiovascular diseases |
| Atchison (2011)^20^ | USA | Cohort  10.3 yrs | N=4501578 (4501578 males) aged 18-100 years | Hospital records | Hospital records | 7515 | Male  RR 0.95 (0.89-1.02) | Age, time, latency, race, number of visits, diagnoses of alcohol-related conditions, obesity and chronic obstructive pulmonary disease |
| Wotton-ORLS1 (2011)^21^ | England | Cohort | N=291462 (159191 males, 132271 females) aged 30 years and above | Hospital records | Hospital records | 1337 | Overall  RR 1.11 (0.89-1.37) | Sex, age, time in single calendar years, district of residence |
| Wotton-ORLS2 (2011)^21^ | England | Cohort | N=192894 (102467 males, 90427 females) aged 30 years and above | Hospital records | Hospital records | 230 | Overall  RR 2.05 (1.30-3.10) | Sex, age, time in single calendar years, district of residence |
| Lin (2011)^22^ | USA | Cohort  8.0 yrs | N=469448 (280883 males and 188565 females) aged 50-71 years | Self-report | Cancer registry | 731 | Male  HR 1.52 (1.21-1.92)^a^  Female  HR 1.62 (0.98-2.68)^a^  Cardia  HR 1.89 (1.43-2.50)  Non-Cardia  HR 0.98 (0.70-1.37) | Age, sex, calories, alcohol, smoking, fruit and vegetable consumption, ethnicity, education, physical activity |
| Carstensen (2012)^23^ | Denmark | Cohort  15.0 yrs | N=5127813 (2553240 males, 2574573 females)  with no age limitations | Diabetes registry | Cancer registry | 6879 | Male HR 1.28 (1.15-1.43) Female HR 1.34 (1.14-1.58) | Age, calendar time, date of birth |
| Hsieh (2012)^24^ | Taiwan | Cohort | N=739155 aged 20 years or more | Health insurance claims data | Health insurance claims data | 3223 | Overall  OR 0.920 (0.836-1.012) | Sex, age |
| Jiang (2012)^25^ | USA | Case-control | N=1948 (973 males, 336 females) aged 49-70 years | Self-report | Cancer registry | 639 | Overall  OR 1.51 (1.10-2.07)^a^  Cardia  OR 0.96 (0.59-1.55)  Non-cardia  OR 1.47 (1.01-2.15) | Age, sex, race, birthplace, education, smoking, BMI |
| Luo (2013)^26^ | USA | Cohort  10.3 yrs | N=145765 (145765 female) aged 50-79 | Self-report | Self-report, medical record review | 121 | Female  HR 0.75 (0.34-1.65) | Age, ethnicity, education, smoking, physical activity, alcohol, total daily energy intake, percent of daily dietary calories from fat, history of hormone therapy use, non-steroidal anti-inflammatory drug use, history of stomach or duodenal ulcer disease, BMI, waist-to-hip ratio |
| Chen (2013)^27^ | Taiwan | Cohort  5.5 yrs | N=98125 (54675 males, 43450 females) aged 20 years or more | Health insurance claims data | Health insurance claims data | 263 | Overall  HR 0.90 (0.65-1.23)  Male  HR 0.81 (0.53-1.23)  Female  HR 1.04 (0.64-1.70) | Age, sex, gastric polyp, partial gastrectomy, gastric ulcer, pneumoconiosis |
| Tseng (2013)^28^ | Taiwan | Cohort | N=329198 (163524 males, 165674 females) aged 45 years or older | Health insurance data | Health insurance data | 1464 | Overall  OR 1.228 (1.089,1.385) | Age, sex, occupation, living region |
| Dankner (2016)^29^ | Israel | Cohort  10.1 yrs | N= 2186196 (1034074 males, 1152122 females) aged 21-89 years | Health insurance claims data | Cancer registry | 2464 | Male  HR 1.69 (1.50,1.90)  Female  HR 1.90 (1.65-2.19) | Age, socioeconomic status, ethnic group, clinic locality |
| He (2018)^30^ | China | Cohort | N=793795 (414274 males, 379521 females) with no age limitations | Health insurance | Health insurance | 607 | Overall  HR 2.51 (1.59-3.95)  Male  HR 2.09 (1.40-3.11)  Female  HR 2.42 (1.44-4.09) | Age, sex, comorbidities, medications, baseline use of inpatient/outpatient care |
| de Jong (2018)^31^ | United Kingdom | Cohort  5.4 yrs | N=666876 (366594 males, 300282 females) aged 18 years or more | Hospital records | Hospital records | 1017 | Overall  IRR 0.98 (0.86-1.11)  Male  IRR 0.95 (0.82-1.10)  Female  IRR 1.02 (0.81-1.27) | Sex, year of birth, clinic |
| Li (2018)^32^ | China | Case-control | N=1954 (1432 males, 522 females) without age limit | Fasting blood glucose or self-report | Hospital records or pathology | 808 | Overall  OR 1.830 (1.375-2.436)  Male  OR 2.062 (1.410-3.402)  Female  OR 1.808 (1.473-2.380) | Age, smoking, family cancer history |
| Zheng (2019)^33^ | Sweden | Cohort  12.2 yrs | N=111198 (54867 males, 56331 females) without age limitation | Self-report, oral glucose tolerance test, fasting blood glucose | Cancer registry | 219 | Overall  HR 0.77 (0.46-1.29) | Sex, calendar year, BMI, smoking, education |
| Cheung (2019)^34^ | Hong Kong | Cohort  7.1 yrs | N=46460 (22093 males, 24367 females) aged 45 years or more | Hospital records | Hospital records | 153 | Overall  HR 1.67 (1.08-2.58)  Cardia  HR 3.40 (1.45-7.97)  Non-cardia  HR 1.53 (0.84-2.78) | Age at the time of *H. pylori* eradication therapy, sex, smoking, alcohol, history of  gastric and duodenal ulcers, comorbidities, and use of other medications |
| Yang (2020)^35^ | Korea | Cohort  4.4 yrs | N=195312 (117610 males, 77702 females) aged 18 years or older | Self-report, fasting blood glucose | Pathology | 198 | Overall  HR 1.76 (1.04-2.97) | Age, sex, year of visit, center, BMI, smoking, alcohol, physical activity, family cancer history, total cholesterol, HDL cholesterol, triglycerides, hypertension, endoscopic diagnosis of atrophic gastritis and intestinal metaplasia |
| Kim (2020)^36^ | Korea | Cohort  8.6 yrs | N=25709497 (12540261 males, 13169236 females) aged 30 years or more | Health insurance claims data | Health insurance claims data | No report | Overall  HR 1.08 (1.06-1.11)  Male  HR 1.08 (1.06-1.11)  Female  HR 1.09 (1.05-1.13) | Age, sex, income, place, hypertension, hyperlipidemia, chronic liver disease, ischemic heart disease, chronic kidney disease |
| Dabo (2021)^37^ | Stomach Cancer Pooling (StoP) Project:  Italy, Spain, Greece, Russia, China, Japan, USA, Brazil | Case-control | N=18069 (11182 males, 6887 females) with no limitation on age | Self-report | Pathology | 5592 | Overall  OR 1.01 (0.94-1.07)  Male  OR 1.06 (0.97-1.16)  Female  OR 0.94 (0.84-1.05)  Cardia  OR 1.16 (1.02-1.33)  Non-cardia  OR 1.03 (0.95-1.12) | Age, race/ethnicity, sex, BMI, socioeconomic status, smoking, alcohol, fruit/vegetable intake, history of gastric ulcer, study site |
| Song (2022) | Asia Cohort Consortium (ACC):  Japan, Korea, China, India | Cohort  14.7 yrs | N=558981 (279096 males, 279885 females) | Self-report | Residence certificates, death certificates, cancer registry, vital statistic registries, or active follow-up surveys | 8556 | Overall  HR 1.15 (1.06-1.25)  Male  HR 1.12 (1.01-1.23)  Female  HR 1.31 (1.07-1.60)  Cardia  HR 1.17 (0.77-1.78)  Non-cardia  HR 1.14 (1.02-1.28) | Sex, smoking, alcohol, education, BMI |


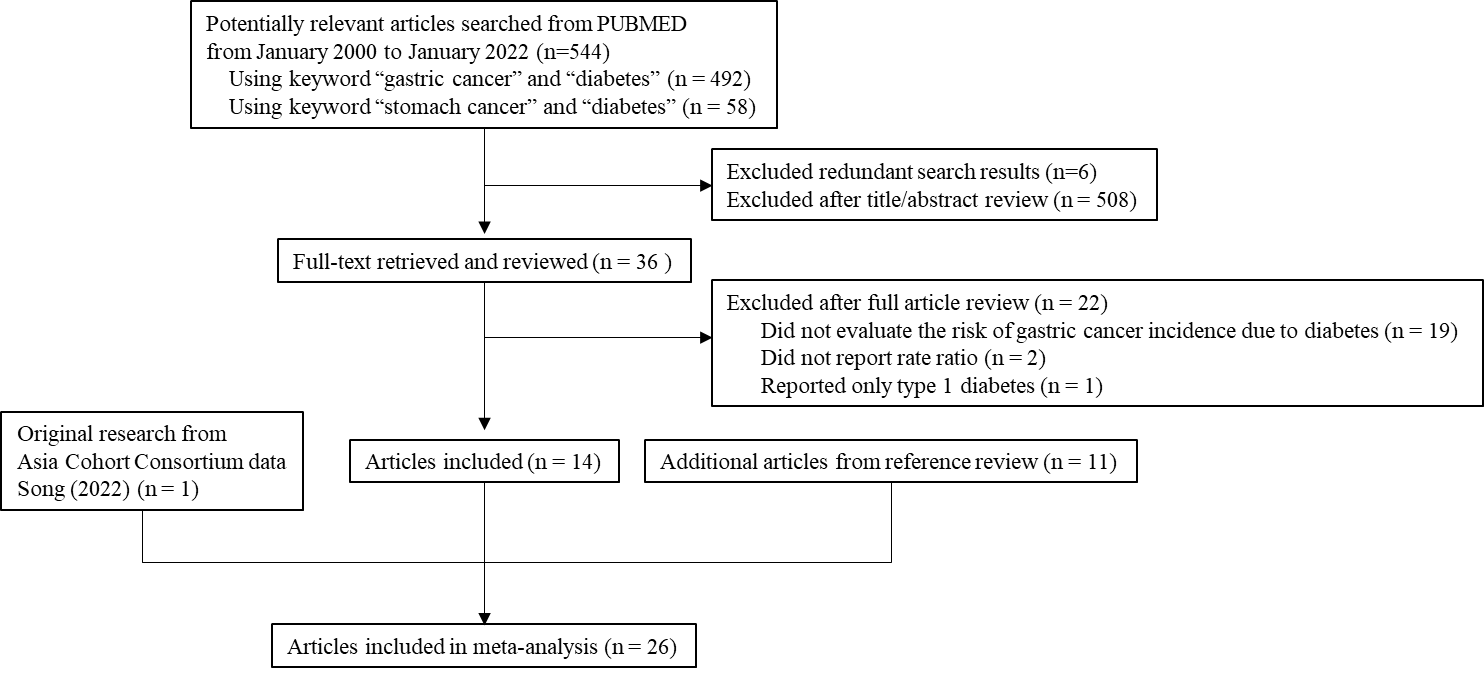


# **Supplementary Figure 3.** Literature search and selection process for the meta-analysis studies on diabetes and gastric cancer incidence

# **Supplementary Table 15.** A meta-analysis of studies on diabetes and gastric cancer incidence


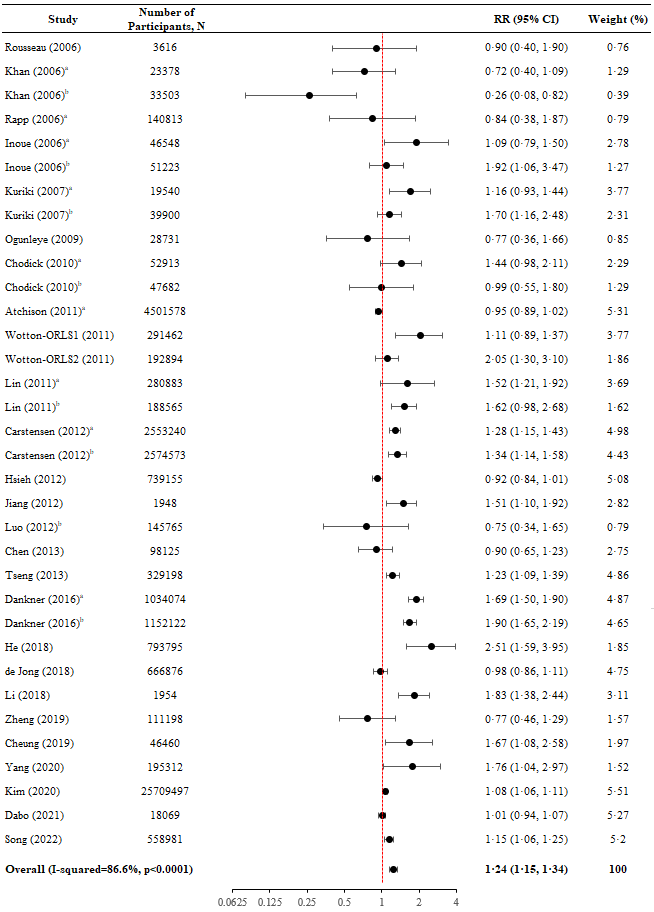


^a^ Study results for males, where the study did not report an overall risk ratio and confidence intervals encompassing males and females

^b^ Study results for females, where the study did not report an overall risk ratio and confidence intervals encompassing males and females

# **Supplementary Table 16.** A subgroup analysis for the meta-analysis of studies on diabetes and gastric cancer association

| **Subgroup** | **No. of RRs** | **Gastric Cancer Incidence** | | | **Between-Subgroup Heterogeneity (*P*)** |
| --- | --- | --- | --- | --- | --- |
|  |  | **RR** | **95% CI** | **I^2^ (%)** |  |
| **Sex** |  |  |  |  |  |
| Male | 16 | 1.19 | 1.09-1.31 | 85.8 | 0.32 |
| Female | 15 | 1.31 | 1.12-1.54 | 88.1 |  |
| **Region** |  |  |  |  |  |
| Asia | 16 | 1.20 | 1.09-1.32 | 78.1 | 0.85 |
| Non-Asia | 20 | 1.22 | 1.07-1.39 | 89.1 |  |
| **Study design** |  |  |  |  |  |
| Cohort | 28 | 1.23 | 1.12-1.34 | 87.7 | 0.48 |
| Prospective | 11 | 1.29 | 1.05-1.59 | 73.9 |  |
| Retrospective | 17 | 1.15 | 1.06-1.23 | 80.9 |  |
| Case-control | 6 | 1.33 | 1.04-1.69 | 81.3 |  |
| **Diabetes ascertainment** |  |  |  |  |  |
| Self-reported | 13 | 1.20 | 1.06-1.37 | 68.2 | 0.68 |
| ICD code or medical record | 18 | 1.24 | 1.12-1.38 | 91.1 |  |
| **Anatomical subsite** |  |  |  |  |  |
| Cardia | 5 | 1.40 | 1.03-1.91 | 75.4 | 0.16 |
| Noncardia | 5 | 1.10 | 0.99-1.23 | 37.8 |  |
| **Study publication year** |  |  |  |  |  |
| 2006-2010 | 11 | 1.12 | 0.91-1.38 | 47.6 | 0.28 |
| 2011-2022 | 23 | 1.26 | 1.16-1.37 | 90.3 |  |
| **Average follow-up time** |  |  |  |  |  |
| < 10 years | 13 | 1.14 | 0.99-1.31 | 63.0 | 0.35 |
| ≥ 10 years | 10 | 1.27 | 1.06-1.52 | 93.2 |  |
| **Covariates** |  |  |  |  |  |
| Adjusted for smoking or alcohol | 19 | 1.21 | 1.08-1.36 | 76.1 | 0.87 |
| Adjusted for BMI or obesity | 17 | 1.16 | 1.04-1.29 | 69.8 |  |
| Adjusted for comorbidities or family history of cancer | 15 | 1.19 | 1.09-1.32 | 78.3 |  |

# **Supplementary Table 17.** A sensitivity analysis using leave-one-out method for the meta-analysis of studies on diabetes and gastric cancer association

|  | **Gastric Cancer Incidence** | | |
| --- | --- | --- | --- |
|  | **RR** | **95% CI** | **% difference^a^** |
| All studies | 1.24 | 1.15-1.34 |  |
| No Rousseau (2006) | 1.24 | 1.15-1.34 | 0.00 |
| No Khan (2006)^b^ | 1.25 | 1.16-1.35 | 0.01 |
| No Khan (2006)^c^ | 1.25 | 1.16-1.35 | 0.01 |
| No Rapp (2006)^b^ | 1.24 | 1.15-1.34 | 0.00 |
| No Inoue (2006)^b^ | 1.24 | 1.15-1.34 | 0.00 |
| No Inoue (2006)^c^ | 1.23 | 1.14-1.33 | -0.01 |
| No Kuriki (2007)^b^ | 1.23 | 1.14-1.33 | -0.01 |
| No Kuriki (2007)^c^ | 1.24 | 1.15-1.34 | 0.00 |
| No Ogunleye (2009) | 1.24 | 1.15-1.34 | 0.00 |
| No Chodick (2010)^b^ | 1.24 | 1.14-1.33 | 0.00 |
| No Chodick (2010)^c^ | 1.24 | 1.15-1.34 | 0.00 |
| No Atchison (2011)^b^ | 1.26 | 1.16-1.37 | 0.02 |
| No Wotton-ORLS1 (2011) | 1.23 | 1.14-1.32 | -0.01 |
| No Wotton-ORLS2 (2011) | 1.25 | 1.15-1.35 | 0.01 |
| No Lin (2011)^b^ | 1.23 | 1.14-1.33 | -0.01 |
| No Lin (2011)^c^ | 1.23 | 1.14-1.33 | -0.01 |
| No Carstensen (2012)^b^ | 1.24 | 1.14-1.34 | 0.00 |
| No Carstensen (2012)^c^ | 1.24 | 1.14-1.34 | 0.00 |
| No Hsieh (2012) | 1.26 | 1.16-1.36 | 0.02 |
| No Jiang (2012) | 1.23 | 1.14-1.33 | -0.01 |
| No Luo (2012)^c^ | 1.24 | 1.15-1.34 | 0.00 |
| No Chen (2013) | 1.25 | 1.16-1.35 | 0.01 |
| No Tseng (2013) | 1.24 | 1.15-1.34 | 0.00 |
| No Dankner (2016)^b^ | 1.22 | 1.13-1.31 | -0.02 |
| No Dankner (2016)^c^ | 1.21 | 1.13-1.30 | -0.02 |
| No He (2018) | 1.22 | 1.13-1.32 | -0.02 |
| No de Jong (2018) | 1.25 | 1.16-1.36 | 0.01 |
| No Li (2018) | 1.22 | 1.13-1.32 | -0.02 |
| No Zheng (2019) | 1.25 | 1.16-1.35 | 0.01 |
| No Cheung (2019) | 1.23 | 1.14-1.33 | -0.01 |
| No Yang (2020) | 1.23 | 1.14-1.33 | -0.01 |
| No Kim (2020) | 1.25 | 1.14-1.38 | 0.01 |
| No Dabo (2021) | 1.26 | 1.16-1.36 | 0.02 |
| No Song (2022) | 1.25 | 1.15-1.35 | 0.01 |

^a^  % difference between HR of analysis excluding the indicated cohort vs. all study analysis

^b^ Study results for males, where the study did not report an overall risk ratio and confidence intervals encompassing males and females

^c^ Study results for females, where the study did not report an overall risk ratio and confidence intervals encompassing males and females

# **Supplementary Figure 4.** A funnel plot for studies on diabetes and gastric cancer incidence

P-values of Begg’s test (*P*=0.56) and Egger’s test (*P*=0.054) suggest no evident publication bias.

# **Supplementary References**

1. Sado J, Kitamura T, Kitamura Y, et al. Rationale, design, and profile of the Three-Prefecture Cohort in Japan: A 15-year follow-up. J Epidemiol 2017;27(4):193-199. DOI: 10.1016/j.je.2016.05.003.

2. Tamakoshi A, Ozasa K, Fujino Y, et al. Cohort Profile of the Japan Collaborative Cohort Study at Final Follow-up. Journal of Epidemiology 2013;23(3):227-232. DOI: 10.2188/jea.je20120161.

3. Tamakoshi A, Yoshimura T, Inaba Y, et al. Profile of the JACC Study. Journal of Epidemiology 2005;15(Supplement_I):S4-S8. DOI: 10.2188/jea.15.s4.

4. Watanabe S, Tsugane S, Sobue T, Konishi M, Baba S. Study Design and Organization of the JPHC Study. Journal of Epidemiology 2001;11(6sup):3-7. DOI: 10.2188/jea.11.6sup_3.

5. Yoo KY, Shin HR, Chang SH, et al. Korean Multi-center Cancer Cohort Study including a Biological Materials Bank (KMCC-I). Asian Pac J Cancer Prev 2002;3(1):85-92. (<https://www.ncbi.nlm.nih.gov/pubmed/12718614>).

6. Oh J-K, Lim MK, Yun EH, et al. Cohort Profile: Community-based prospective cohort from the National Cancer Center, Korea. International Journal of Epidemiology 2015:dyv302. DOI: 10.1093/ije/dyv302.

7. Pednekar MS, Gupta R, Gupta PC. Association of Blood Pressure and Cardiovascular Mortality in India: Mumbai Cohort Study. American Journal of Hypertension 2009;22(10):1076-1084. DOI: 10.1038/ajh.2009.131.

8. Ozasa K, Grant EJ, Kodama K. Japanese Legacy Cohorts: The Life Span Study Atomic Bomb Survivor Cohort and Survivors' Offspring. J Epidemiol 2018;28(4):162-169. DOI: 10.2188/jea.JE20170321.

9. Kim JY, Ko Y-J, Rhee CW, et al. Cardiovascular Health Metrics and All-cause and Cardiovascular Disease Mortality Among Middle-aged Men in Korea: The Seoul Male Cohort Study. Journal of Preventive Medicine & Public Health 2013;46(6):319-328. DOI: 10.3961/jpmph.2013.46.6.319.

10. Shu X-O, Li H, Yang G, et al. Cohort Profile: The Shanghai Men’s Health Study. International Journal of Epidemiology 2015;44(3):810-818. DOI: 10.1093/ije/dyv013.

11. Zheng W, Chow W-H, Yang G, et al. The Shanghai Women's Health Study: Rationale, Study Design, and Baseline Characteristics. American Journal of Epidemiology 2005;162(11):1123-1131. DOI: 10.1093/aje/kwi322.

12. Yamakawa M, Wada K, Goto Y, et al. Associations between coffee consumption and all-cause and cause-specific mortality in a Japanese city: the Takayama study. Public Health Nutrition 2019;22(14):2561-2568. DOI: 10.1017/s1368980019000764.

13. Rousseau MC, Parent ME, Pollak MN, Siemiatycki J. Diabetes mellitus and cancer risk in a population-based case-control study among men from Montreal, Canada. Int J Cancer 2006;118(8):2105-9. (In eng). DOI: 10.1002/ijc.21600.

14. Khan M, Mori M, Fujino Y, et al. Site-specific cancer risk due to diabetes mellitus history: evidence from the Japan Collaborative Cohort (JACC) Study. Asian Pac J Cancer Prev 2006;7(2):253-9. (In eng).

15. Rapp K, Schroeder J, Klenk J, et al. Fasting blood glucose and cancer risk in a cohort of more than 140,000 adults in Austria. Diabetologia 2006;49(5):945-52. (In eng). DOI: 10.1007/s00125-006-0207-6.

16. Inoue M, Iwasaki M, Otani T, Sasazuki S, Noda M, Tsugane S. Diabetes mellitus and the risk of cancer: results from a large-scale population-based cohort study in Japan. Arch Intern Med 2006;166(17):1871-7. (In eng). DOI: 10.1001/archinte.166.17.1871.

17. Kuriki K, Hirose K, Tajima K. Diabetes and cancer risk for all and specific sites among Japanese men and women. Eur J Cancer Prev 2007;16(1):83-9. (In eng). DOI: 10.1097/01.cej.0000228404.37858.40.

18. Ogunleye AA, Ogston SA, Morris AD, Evans JM. A cohort study of the risk of cancer associated with type 2 diabetes. Br J Cancer 2009;101(7):1199-201. (In eng). DOI: 10.1038/sj.bjc.6605240.

19. Chodick G, Heymann AD, Rosenmann L, et al. Diabetes and risk of incident cancer: a large population-based cohort study in Israel. Cancer Causes Control 2010;21(6):879-87. (In eng). DOI: 10.1007/s10552-010-9515-8.

20. Atchison EA, Gridley G, Carreon JD, Leitzmann MF, McGlynn KA. Risk of cancer in a large cohort of U.S. veterans with diabetes. Int J Cancer 2011;128(3):635-43. (In eng). DOI: 10.1002/ijc.25362.

21. Wotton CJ, Yeates DG, Goldacre MJ. Cancer in patients admitted to hospital with diabetes mellitus aged 30 years and over: record linkage studies. Diabetologia 2011;54(3):527-34. (In eng). DOI: 10.1007/s00125-010-1987-2.

22. Lin SW, Freedman ND, Hollenbeck AR, Schatzkin A, Abnet CC. Prospective study of self-reported diabetes and risk of upper gastrointestinal cancers. Cancer Epidemiol Biomarkers Prev 2011;20(5):954-61. (In eng). DOI: 10.1158/1055-9965.Epi-10-1244.

23. Carstensen B, Witte DR, Friis S. Cancer occurrence in Danish diabetic patients: duration and insulin effects. Diabetologia 2012;55(4):948-58. (In eng). DOI: 10.1007/s00125-011-2381-4.

24. Hsieh MC, Lee TC, Cheng SM, Tu ST, Yen MH, Tseng CH. The influence of type 2 diabetes and glucose-lowering therapies on cancer risk in the Taiwanese. Exp Diabetes Res 2012;2012:413782. (In eng). DOI: 10.1155/2012/413782.

25. Jiang X, Bernstein L, Tseng CC, Wu AH. Diabetes and risk of esophageal and gastric adenocarcinomas. Int J Cancer 2012;131(6):1417-22. (In eng). DOI: 10.1002/ijc.27390.

26. Luo J, Chlebowski R, Liu S, et al. Diabetes mellitus as a risk factor for gastrointestinal cancers among postmenopausal women. Cancer Causes Control 2013;24(3):577-85. (In eng). DOI: 10.1007/s10552-012-9996-8.

27. Chen YL, Cheng KC, Lai SW, et al. Diabetes and risk of subsequent gastric cancer: a population-based cohort study in Taiwan. Gastric Cancer 2013;16(3):389-96. (In eng). DOI: 10.1007/s10120-012-0197-7.

28. Tseng CH. Diabetes, insulin use, and gastric cancer: a population-based analysis of the Taiwanese. J Clin Gastroenterol 2013;47(6):e60-4. (In eng). DOI: 10.1097/MCG.0b013e31827245eb.

29. Dankner R, Boffetta P, Balicer RD, et al. Time-Dependent Risk of Cancer After a Diabetes Diagnosis in a Cohort of 2.3 Million Adults. Am J Epidemiol 2016;183(12):1098-106. (In eng). DOI: 10.1093/aje/kwv290.

30. He X, Shi L, Wu J. Retrospective database analysis of cancer risk in patients with type 2 diabetes mellitus in China. Curr Med Res Opin 2018;34(6):1089-1098. (In eng). DOI: 10.1080/03007995.2017.1421527.

31. de Jong R, Peeters P, Burden AM, et al. Gastrointestinal cancer incidence in type 2 diabetes mellitus; results from a large population-based cohort study in the UK. Cancer Epidemiol 2018;54:104-111. (In eng). DOI: 10.1016/j.canep.2018.04.008.

32. Li F, Du H, Li S, Liu J. The Association Between Metabolic Syndrome and Gastric Cancer in Chinese. Front Oncol 2018;8:326. (In eng). DOI: 10.3389/fonc.2018.00326.

33. Zheng J, Rutegård M, Santoni G, et al. Prediabetes and diabetes in relation to risk of gastric adenocarcinoma. Br J Cancer 2019;120(12):1147-1152. (In eng). DOI: 10.1038/s41416-019-0470-1.

34. Cheung KS, Chan EW, Chen L, Seto WK, Wong ICK, Leung WK. Diabetes Increases Risk of Gastric Cancer After Helicobacter pylori Eradication: A Territory-Wide Study With Propensity Score Analysis. Diabetes Care 2019;42(9):1769-1775. (In eng). DOI: 10.2337/dc19-0437.

35. Yang HJ, Kang D, Chang Y, et al. Diabetes mellitus is associated with an increased risk of gastric cancer: a cohort study. Gastric Cancer 2020;23(3):382-390. (In eng). DOI: 10.1007/s10120-019-01033-8.

36. Kim SK, Jang JY, Kim DL, et al. Site-specific cancer risk in patients with type 2 diabetes: a nationwide population-based cohort study in Korea. Korean J Intern Med 2020;35(3):641-651. (In eng). DOI: 10.3904/kjim.2017.402.

37. Dabo B, Pelucchi C, Rota M, et al. The association between diabetes and gastric cancer: results from the Stomach Cancer Pooling Project Consortium. Eur J Cancer Prev 2021 (In eng). DOI: 10.1097/cej.0000000000000703.
